# Optogenetic engineering of BAX to control mitochondrial permeabilization and attenuate apoptosis in cells

**DOI:** 10.1038/s12276-025-01605-y

**Published:** 2025-12-26

**Authors:** Dain Lee, Hyunjun Bae, Dongwoo Oh, Minseop Kim, Ju-Hee Kim, Jinchul Ahn, Seok-Hyeon Kang, Seo-Hee You, Dong-Hwee Kim, Hyun Jeong Oh, Won Do Heo, Seok Chung

**Affiliations:** 1 KU-KIST Graduate School of Converging Science and Technology, Korea University, Seoul, Republic of Korea; 2 School of Mechanical Engineering, Korea University, Seoul, Republic of Korea; 3 Department of Mechanical Engineering, Stanford University, Stanford, CA USA; 4 Center for Advanced Biomolecular Recognition, Korea Institute of Science and Technology, Seoul, Republic of Korea; 5 Department of Integrative Energy Engineering, College of Engineering, Korea University, Seoul, Republic of Korea; 6 Biomaterials Research Center, Biomedical Research Division, Korea Institute of Science and Technology, Seoul, Republic of Korea; 7 Department of Biological Sciences, Korea Advanced Institute of Science and Technology, Daejeon, Republic of Korea; 8 Center for Brain Technology, Brain Science Institute, Korea Institute of Science and Technology, Seoul, Republic of Korea

**Keywords:** Biological techniques, Expression systems

## Abstract

Although considerable research has focused on enhancing the apoptotic function of BAX for several decades, inhibition of its functionality remains relatively underexplored, despite intensive BAX activation occurring in various neurodegenerative diseases. Here we present a protein engineering approach to modulate BAX integration into the mitochondrial outer membrane, establishing a tunable strategy for antiapoptosis. Utilizing optogenetic methods that employ cryptochrome 2 and its binding partner cryptochrome-interacting basic helix loop helix 1, we achieved precise spatial control over BAX localization, a critical determinant of its function. Our results demonstrate that the engineered BAX variant is effectively incapacitated in its apoptotic function while also modulating endogenous BAX activity to enhance cellular resistance to apoptosis. These findings not only advance our understanding of BAX regulation but also offer promising prospects for the development of therapeutic strategies against apoptosis-related diseases.

## Engineered BAX modulates apoptosis for neuroprotection

BAX is a protein that helps control cell death, which is important for removing damaged or harmful cells. However, when this process goes wrong, it can lead to diseases such as cancer or Alzheimer’s disease. Researchers explored a new way to control BAX using light to better understand and potentially treat these conditions. The study used optogenetics to precisely manipulate BAX in cells. They created a modified version of BAX that can be controlled with blue light, allowing them to study its effects on cell death. This approach helps avoid unintended side effects common with other methods. Researchers found that their modified BAX could reduce cell death by preventing BAX from forming pores in the cell’s mitochondria. This discovery suggests that controlling BAX with light could be a promising strategy for treating diseases.

This summary was initially drafted using artificial intelligence, then revised and fact-checked by the author.

## Introduction

BAX belongs to the BCL-2 family, a group of proteins that plays a crucial role in governing apoptosis, a highly regulated process designed to eliminate damaged, unnecessary or potentially harmful cells in the body^1^. In conjunction with its counterpart BCL-2 and other related proteins, BAX participates in the regulation of mitochondrial outer membrane permeabilization (MOMP), which is a critical step in the apoptotic pathway^2^.

BAX is a proapoptotic protein that possesses BCL-2 homology (BH) domains, which are critical for protein-protein interactions within the BCL-2 family^3–5^. Upon receiving apoptosis signals, BAX undergoes a conformational change that enables its insertion into the mitochondrial outer membrane (MOM). Upon integration, BAX oligomerizes by interacting with other BAX molecules through their BH domains and creates pores in the MOM, facilitating the release of cytochrome c (CytC) and other apoptotic factors from the mitochondria into the cytoplasm. The subsequent release of CytC initiates a series of events resulting in cell death^6^. The delicate equilibrium between proapoptotic proteins such as BAX and antiapoptotic proteins such as BCL-2 is fundamental for determining whether a cell will undergo apoptosis or survive. In healthy cells, these proteins coexist in a finely tuned balance; however, various signals and stress conditions can disrupt this equilibrium, tilting the scales toward apoptosis^7,8^. Dysregulation of the apoptotic pathway, including abnormalities in BAX expression or function, has been associated with various diseases including cancer, autoimmune diseases, cardiovascular diseases, aging and neurodegenerative disorders^9–15^.

Owing to its involvement in apoptosis, BAX, along with other BCL-2 family proteins, has been investigated as a potential target for therapeutic interventions, particularly in cancer treatment^16–18^. However, most previous research has focused on proapoptotic induction using BAX for disease treatment, whereas antiapoptosis is considered crucial in diseases induced by excessive cell death, such as Alzheimer’s disease or Parkinson’s disease^19–21^. This study aims to explore the antiapoptotic function of a modified BAX protein, using optogenetics for precise control, to achieve a comprehensive understanding of the structural intricacies and functional manipulation underlying BAX activity.

Optogenetics is a powerful technique that uses light to control cellular activities in living organisms by employing light-sensitive proteins often derived from microbial organisms such as algae or bacteria^22^. In the context of studying BAX, optogenetics offers several distinct advantages including spatial precision, reversibility and a reduction in off-target effects. The spatial precision of optogenetic tools permits the targeted control of BAX activity in specific cellular compartments, providing insights into its localized effects within the mitochondrial function in this study. The reversible nature of optogenetic control enables iterative modulation of BAX activity, which allows the investigation of transient changes and helps differentiate between acute and prolonged cellular responses. Unlike traditional genetic manipulation techniques that can accidentally impact multiple cellular processes, optogenetics provides a precise and direct method for protein control that minimizes unintended side effects.

**Fig. 1:**
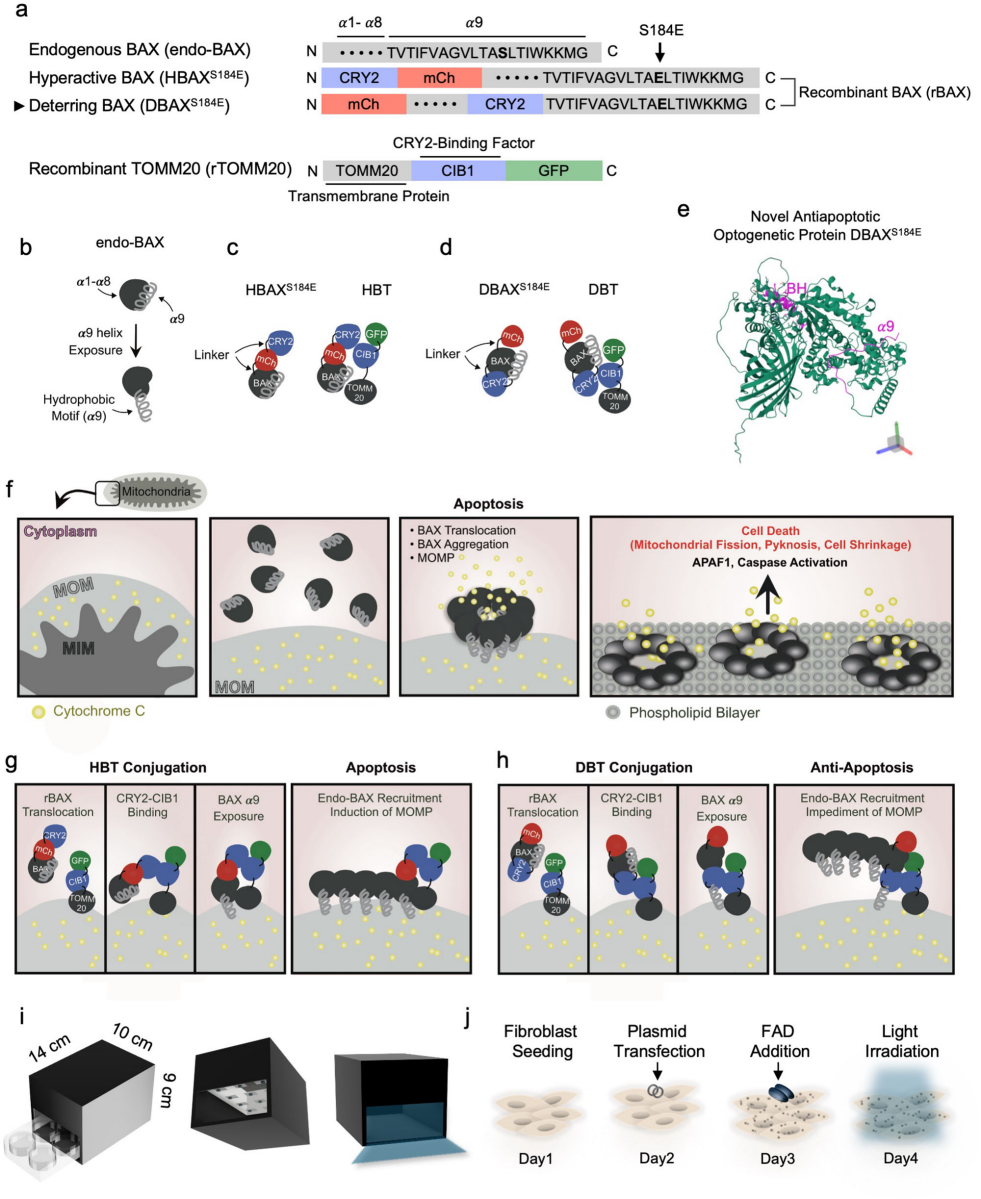
Design and working principles of endo-BAX and engineered BAX variants. **a** A schematic depiction of the functional domains in endo-BAX, rBAX and recombinant TOMM20 (rTOMM20). In hyperactive BAX (HBAX^S184E^), the residue at position 184 in the *α*9 domain is mutated to glutamic acid, and the resulting construct is fused to CRY2 and mCherry (mCh). Deterring BAX (DBAX^S184E^) is a novel BAX variant developed in this study; it also carries the S184E mutation but differs by having CRY2 inserted between the *α*8 and *α*9 domains, in addition to an mCh fusion. rTOMM20 is fused to CIB1, as well as to GFP. All recombinant proteins contain nonfunctional linker sequences. **b** An illustration of endo-BAX with its nine *α*-helical segments. The C-terminal *α*9 helix contains a hydrophobic motif. **c** Optogenetically modified constructs in the HBT system. HBAX^S184E^ and rTOMM20 are shown along with their proposed interaction. **d** Optogenetically modified constructs in the DBT system. DBAX^S184E^ and rTOMM20 are illustrated, including their predicted interaction. **e** The predicted PDB structure of DBAX^S184E^. **f** Model of mitochondrial membranes (outer, MOM; inner, mitochondrial inner membrane (MIM)), with CytC in the intermembrane space and endo-BAX in the cytoplasm. Upon receiving apoptotic signals, the *α*9 helix of BAX is exposed, allowing BAX insertion into the MOM, aggregation and subsequent release of CytC into the cytosol, which triggers apoptotic signaling. **g** Mechanism of HBT complex formation under blue light stimulation. HBAX^S184E^ translocates to the MOM, where CRY2 binds to CIB1 in rTOMM20. The exposed *α*9 helix of HBAX^S184E^ inserts into the MOM, recruiting endo-BAX and inducing MOMP. **h** Mechanism of DBT complex formation upon optogenetic stimulation. DBAX^S184E^ also translocates to the MOM and binds rTOMM20 via CRY2–CIB1 interaction. Although its *α*9 helix is inserted into the MOM, the binding of CRY2–CIB1 above the hydrophobic motif in DBAX^S184E^ disrupts BAX oligomerization, limiting MOMP. **i** A schematic of the blue light irradiation chamber and calibration. The light chamber is depicted with a 4 × 9 cm² entrance, accommodating a standard six-well cell culture plate. **j** Workflow for recombinant protein expression in cells. See also Supplementary Figs. 1 and 2 and Supplementary Table 1 for more details.

In this study, optogenetic control was employed to translocate cytoplasmic BAX into mitochondria using cryptochrome 2 (CRY2) and its binding partner cryptochrome-interacting basic helix-loop-helix 1 (CIB1), which are reversibly associated under blue light and dissociated in the dark^23,24^. For genetic modification, CRY2 was fused to BAX, which localizes in the cytoplasm in the dark owing to its point mutation (S184E), while CIB1 was fused to TOMM20, a protein resident in the MOM^25^. In detail, CRY2 was inserted between the BAX *α*8 and *α*9 motifs to deter collective BAX insertion into the MOM by weakening the anchor, the hydrophobic motif located on the *α*9 helix, and also moving the BH domain binding site away from the membrane^26,27^. The final construct consisted of mCherry::BAX *α*1–*α*8::CRY2::*α*9^S184E^ (Fig. 1a, Supplementary Fig. 1 and Supplementary Table 1). This novel antiapoptotic optogenetic system was validated by comparing its effects on cellular compartments, such as the mitochondria and nucleus, with those of the proapoptotic optogenetic system of BAX, consisting of CRY2::mCherry::BAX^S184E^. For simplicity, the newly developed antiapoptotic recombinant protein unit was named deterring BAX-TOMM20 (DBT), whereas the proapoptotic recombinant protein unit, designed based on previous literature, was named hyperactive BAX-TOMM20 (HBT)^28^.

## Materials and methods

### Plasmid constructs

For transient transfection of either the HBT or the DBT system, recombinant TOMM20 (rTOMM20) plasmid was purchased from Addgene (#226667). Information regarding the custom-designed recombinant BAX (rBAX) sequences is provided in Supplementary Table 1.

### Light chamber fabrication

A light chamber was three-dimensional (3D)-printed using polyethylene filaments and designed with dimensions of 10 cm × 14 cm × 9 cm (width × length × height). The plate entrance was designed to be 9 cm × 4 cm (width × height) with a door that could open or close the chamber entrance. A blue printed circuit board light-emitting diode (DC 5 V, 2.16 W, 60 mA, 120° light angle, #2835, LG Innotek) was installed on the light-emitting diode board attached to the ceiling of the light chamber. In addition, a C-type charging socket, switch, and ventilation wickets were installed on the backside of the chamber. The battery could sustain the light chamber for 4 h when fully charged. See Supplementary Fig. 2 for detailed illustrations.

### Cell culture and transient transfection

**Fig. 2:**
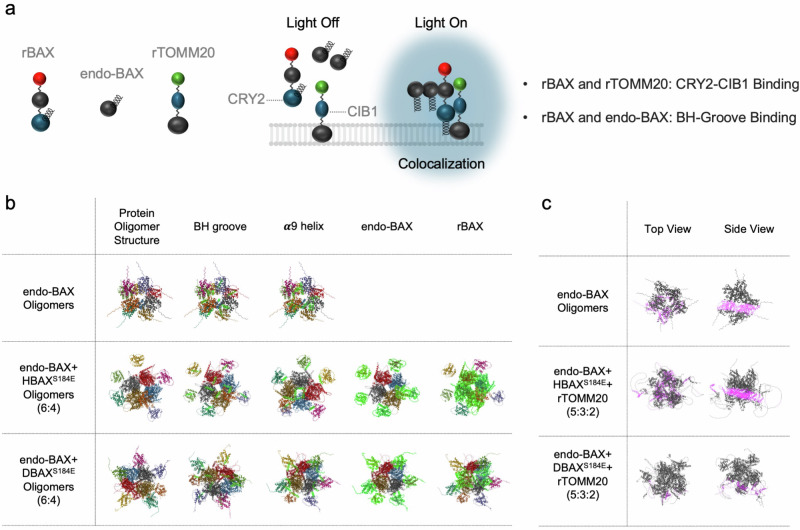
Simulations of protein aggregation. **a** A schematic representation of rBAX, endo-BAX and rTOMM20 interactions. Under dark conditions, the three components remain dissociated. Upon blue light exposure, however, they assemble at the MOM. In this system, rBAX and rTOMM20 interact via CRY2–CIB1 binding, whereas rBAX and endo-BAX associate through their BH grooves. **b** To model optogenetically induced BAX aggregation, AlphaFold Multimer simulations were performed using either HBAX^S184E^ or DBAX^S184E^ at a 6:4 ratio to endo-BAX (total of ten proteins). Each protein is rendered in a different color, with BH grooves, α9 helices, endo-BAX or rBAX highlighted in green. In contrast to endo-BAX and HBAX^S184E^, the DBAX^S184E^ complex exhibits minimal pore formation. **c** To simulate conditions in which rTOMM20 associates with rBAX under blue light irradiation, endo-BAX, rBAX and rTOMM20 were combined in a 5:3:2 ratio. Membrane-embedded residues are highlighted in pink, and the remnants are shown in gray. Both the top and side views are presented. The DBAX^S184E^–rTOMM20 (DBT) complex displays limited hydrophobic motif insertion into the MOM compared to the HBAX^S184E^–rTOMM20 (HBT) complex.

**Fig. 3:**
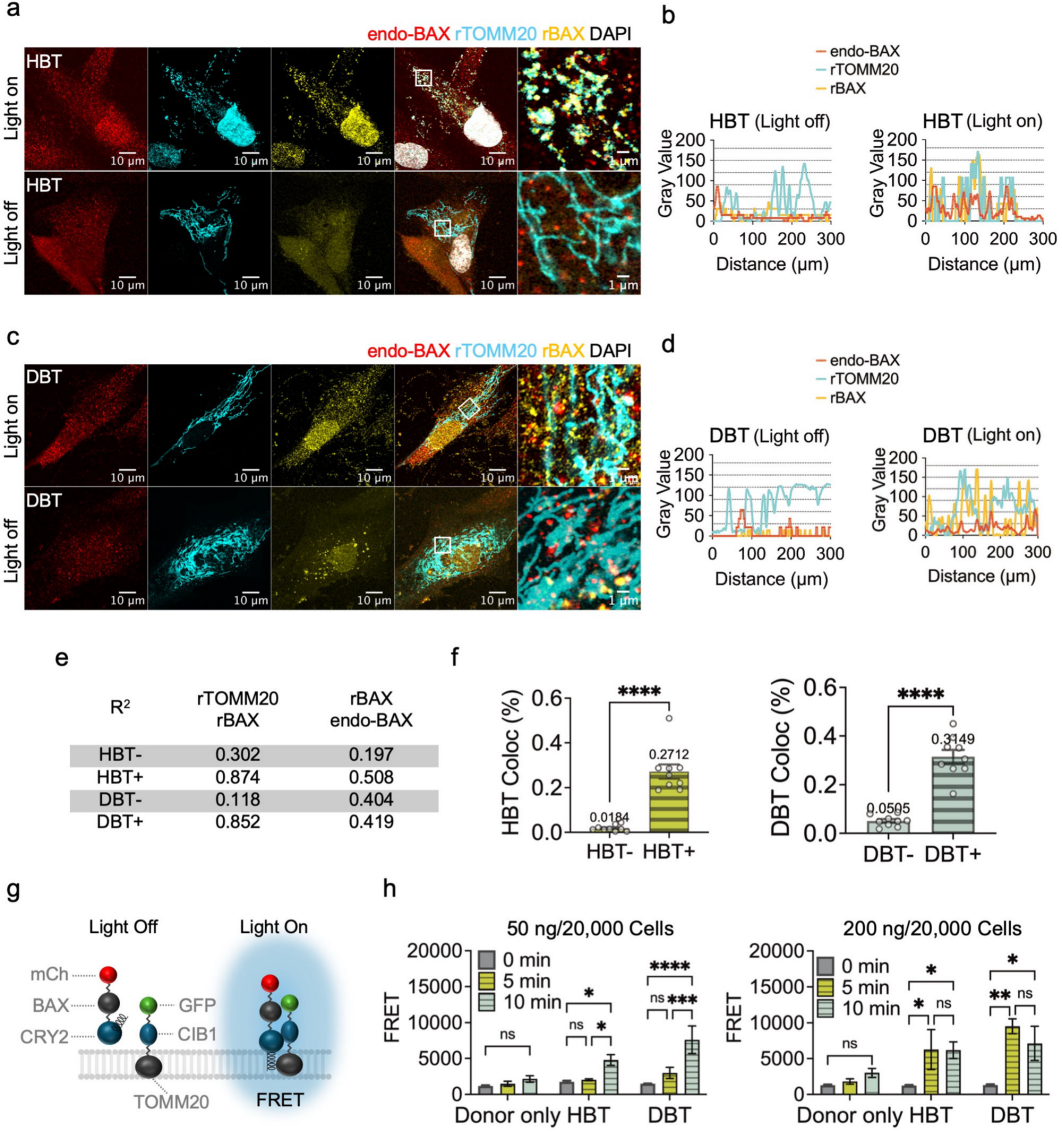
Optogenetic control of DBT and its impact on endo-BAX. **a**, **c** Representative immunofluorescence images illustrating the colocalization of endo-BAX, rTOMM20 and rBAX in cells transfected with either the HBT (**a**) or DBT (**c**) complex and subjected to blue light irradiation. The images are shown under conditions of continuous blue light exposure (‘Light On’) or in the absence of light (‘Light Off’). b, d The intensity profiles corresponding to the boxed regions in the microscopy images for **a** (**b**) and **c** (**d**). The red, cyan and yellow lines represent the intensities of endo-BAX, rTOMM20 and rBAX, respectively. **e** The *R*² values (coefficient of determination) from simple linear regression analyses of rTOMM20–rBAX and rBAX–endo-BAX interactions in HBT or DBT conditions under dark and blue light conditions (n = 5 per group). **f** Bar graphs showing the colocalization ratio (Coloc%) between rTOMM20 and rBAX in cells transfected with HBT or DBT (n = 6 per group). The negative sign indicates nonirradiated cells, and the positive sign indicates blue-light-irradiated cells. Statistical significance was determined by unpaired t-tests (****P < 0.0001). **g** A schematic of rBAX within the DBT unit. Upon blue light irradiation, DBT units associate, facilitating interactions between mCherry (mCh) and GFP, which enables FRET. **h** A quantification of FRET efficiency between the GFP donor and mCh acceptor, showing increased efficiency under prolonged blue light irradiation (n = 6 per group). The data were analyzed by two-way ANOVA with Tukey’s multiple comparisons test. *P < 0.05; **P < 0.01; ***P < 0.001; ****P < 0.0001; ns, not significant; mean ± s.e.m. for all data. See also Supplementary Figs. 8 and 9 for more details.

Human dermal fibroblast-neonatal (NHDF-Neo) cells (#CC-2509, Lonza) were maintained in a culture medium consisting of RPMI-1640 (#10-040-CV, Cellgro), 10% fetal bovine serum (FBS, #A2720803, Gibco, Thermo Fisher Scientific) and 1% penicillin–streptomycin (#15140163, Invitrogen, Thermo Fisher Scientific) in a 37 °C humidity-controlled incubator with 5% CO_2_. Lipofectamine 3000 (#L3000015; Thermo Fisher Scientific) was used as the transfection reagent. Initially, 2 × 10^5^ cells were plated in each well of a six-well plate (#30006, SPL Life Sciences) and incubated for 24 h at 37 °C. The cell medium was then aspirated, and FBS-reduced medium (RPMI-1640, 2% FBS and 1% penicillin–streptomycin) was added an hour before transfection. P3000 reagent (2 μl) was mixed with 1 μg of the HBT or DBT plasmids (500 ng rBAX and 500 ng rTOMM20 (1:1 ratio; Supplementary Fig. 3) in a total volume of 1 μl) in 122 μl of Opti-MEM (#31985070, Gibco, Thermo Fisher Scientific). For the control condition, the reagent solution was mixed with 1 μg rTOMM20 plasmids (1 μl) instead. Separately, 4 μl of Lipofectamine 3000 was added to 121 μl of Opti-MEM per well of the six-well plate. After 5 min incubation at room temperature, the mixtures were combined to a total volume of 250 μl and incubated at room temperature for 30 min. Subsequently, 200 μl of the incubated mixture was added dropwise to each well. The medium was aspirated and replaced with fresh culture medium containing 5 μM flavin adenine dinucleotide disodium salt hydrate (FAD, #F6625, Sigma-Aldrich) 24 h after transfection. For further experiments, the cells were illuminated with blue light 48 h post transfection in the light chamber for 20 min (if not otherwise mentioned in the text; Fig. 2a–f, Fig. 3, Supplementary Figs. 4 and 5).

### FRET assay

Cells at 48 h post transfection with either 50 or 200 ng of HBT or DBT plasmids per 20,000 cells in a 96-well plate were serially irradiated with blue light for 0, 5 or 10 min. The cells were then immediately analyzed using Förster resonance energy transfer (FRET) analysis in a fluorescence-detectable multimode microplate reader (Hidex Sense, HIDEX Oy).

### Immunofluorescence microscopy

A total of 24 h post transfection, the transfected cells were dissociated from the surface using 0.25% trypsin (#24200-072, Thermo Fisher Scientific) and plated on cell culture slide I (#30408, SPL Life Sciences) at a density of 5 × 10^4^ cells per well. The cells were incubated in a 37 °C humidity-controlled incubator with 5% CO_2_ for 24 h. For immunofluorescence microscopy sample preparation, the cells were irradiated with blue light for 20 min and immediately prepared for the next step in most analyses (1-min incubation in the dark) or incubated in the dark for 10 min. In drug-treated experiments, blue light was applied for 10 min (Supplementary Fig. 6). Following incubation in the dark, the cells were fixed with 4% paraformaldehyde solution (#PC2031-100-00, Biosesang) and stored at 4 °C overnight. The fixed cells were treated with 0.4% Triton X-100 in PBS for 20 min at room temperature. After aspirating the buffer, cells were treated with 5% bovine serum albumin in PBS for 30 min at room temperature. The buffer was then aspirated, and the cells were incubated with primary antibodies in a mixture of 0.2% Tween in PBS (PBST) and 5% bovine serum albumin (1:1 ratio) overnight at 4 °C. The primary antibodies were aspirated, and the cells were washed thrice with PBST for 5 min at room temperature. The cells were then incubated with secondary antibodies for 1 h at room temperature. After aspirating the secondary antibodies, the cells were washed thrice with PBST for 5 min at room temperature. The mounting medium was applied dropwise to the samples, which were mounted on a 24 mm × 60 mm microscope cover glass (#HSU-0101242, Marienfeld). The samples were examined under a fluorescence microscope (Axio Imager M1; Carl Zeiss AG) or LSM 900 confocal microscope (LSM 900; Carl Zeiss AG) (see Supplementary Table 2 for antibody details).

### Live-cell imaging

Transfected cells were treated with 200 μM cisplatin and maintained in a live-cell imaging system (Incubator TS, Live Cell Instrument) to ensure a humidity-controlled environment at 37 °C with 5% CO_2_. The cells were visualized using the CelenaX imaging system (CELENA X, Logos Biosystems). Time-lapse imaging was conducted by irradiating cells with blue light for 1,000 ms every 5 min during a 3 h recording period. Translocation events of mCherry-tagged rBAX and green fluorescent protein (GFP)-tagged rTOMM20 were visualized and traced under a microscope.

### Protein structure simulation

For predicting recombinant protein oligomer structures, the AlphaFold online tool (developed by DeepMind, with data from EMBL-EBI) was used for multimer binding structure prediction and creation of PDB files. RCSB PDB (managed by Rutgers University and University of California San Diego, supported by the NSF, NIH and DOE) was utilized for visualizing the multimer structures^29,30^.

### Statistical analysis

Colocalization and morphological analyses were conducted using ImageJ software (NIH). For the colocalization assessment, the color-merged immunostained fluorescence images of the red and green (or blue) channels were thresholded using ImageJ software. The ratio of the colocalized area to the red-thresholded area was analyzed. Alternatively, the plot profile plugin in ImageJ was employed for both colocalization assessment and fluorescence intensity measurement. For morphological analysis, the immunostained fluorescence images were thresholded, and size filtering was performed. The morphological analysis of subcellular compartments was conducted by setting subcellular regions of interest (ROIs, randomly selected) in multiple single-cell images. Colocalization, CytC and CC3 assays were performed on a per-cell basis, whereas APAF1 analysis was conducted for each image obtained from the slide samples. All experiments were performed three times independently. Quantitative data values were analyzed using Prism software (GraphPad Software), and the bar graphs were presented as the mean ± standard error of the mean. Two-tailed tests were conducted for every statistical analysis (95% confidence).

## Results

### Characterization of custom-built recombinant proteins

In this study, we aimed to manipulate the MOMPs induced by endogenous BAX (endo-BAX), which have a distinct structure comprising *α*1–*α*9, with *α*9 containing a hydrophobic motif (Fig. 1a, b). To achieve precise control of optogenetic BAX for proapoptotic induction, in comparison with DBT, this study introduced HBT, which demonstrates stable and controllable insertion of CRY2-fused BAX into the MOM upon blue light activation^28^. In the recombinant protein constructs, BAX was fused to CRY2 and mCherry in distinct configurations for HBAX^S184E^ and DBAX^S184E^ (Fig. 1a, c, d). In HBAX^S184E^, full-length CRY2 and mCherry were positioned upstream of the complete BAX sequence, incorporating a point mutation at residue 184 (S184E) (Supplementary Fig. 7). By contrast, DBAX^S184E^ also placed the full mCherry sequence upstream but introduced CRY2 between the *α*8 and *α*9 helices of BAX. As an optogenetic binding partner, TOMM20–CIB1–GFP (recombinant TOMM20; rTOMM20) was engineered to facilitate the translocation of BAX from the cytosol to the mitochondria. Therefore, the novelty of this study is embodied in the engineered DBAX^S184E^ structure, in which the binding of CRY2 and CIB1 immediately above the membrane hinders MOMP because the anchoring function of the hydrophobic motif within the *α*9 helices is limited and BH groove binding, which is essential for MOMP, is deterred, thereby modulating proapoptotic activity (Fig. 1e).

The sequential process of MOMP induced by endo-BAX is as follows. In the normal state, CytC is retained within the mitochondria by the intact MOM, whereas BAX remains in the cytoplasm^6^. During apoptosis, BAX translocates to the MOM, accompanied by the exposure of its *α*9 helix. Another BAX aligns next to the first on MOM by generating junctions via BH grooves, leading to pore formation due to extensive BAX insertion^3–5^. This process results in CytC leakage into the cytosol, triggering cell death via activation of APAF1 and caspase proteins^31^. It is accompanied by morphological changes such as mitochondrial fission, nuclear pyknosis and cell shrinkage^32,33^ (Fig. 1f).

Apoptosis by HBT is induced by the binding of CRY2 in rBAX with its binding partner CIB1, which is part of rTOMM20, thereby facilitating vast endo-BAX recruitment to the MOM and inducing MOMP (Fig. 1g). In comparison, rBAX of DBT, which exhibits limited insertion into the MOM along with endo-BAX, hinders the MOMP under blue light irradiation (Fig. 1h).

To verify the antiapoptotic functionality of the DBT, a blue light chamber was specifically generated to fit into six-well cell culture plates (Fig. 1i and Supplementary Fig. 2). For live-cell imaging, a 488 nm blue laser equipped with a fluorescence microscope was utilized. For analysis, cells were transfected with plasmids 24 h after seeding and FAD was added to the culture medium 24 h post transfection. Finally, the cells were irradiated with blue light at 48 h post transfection (Fig. 1j).

### Aggregation simulation and microscopic analysis of DBT reveal attenuated pore formation impacting endo-BAX

**Fig. 4:**
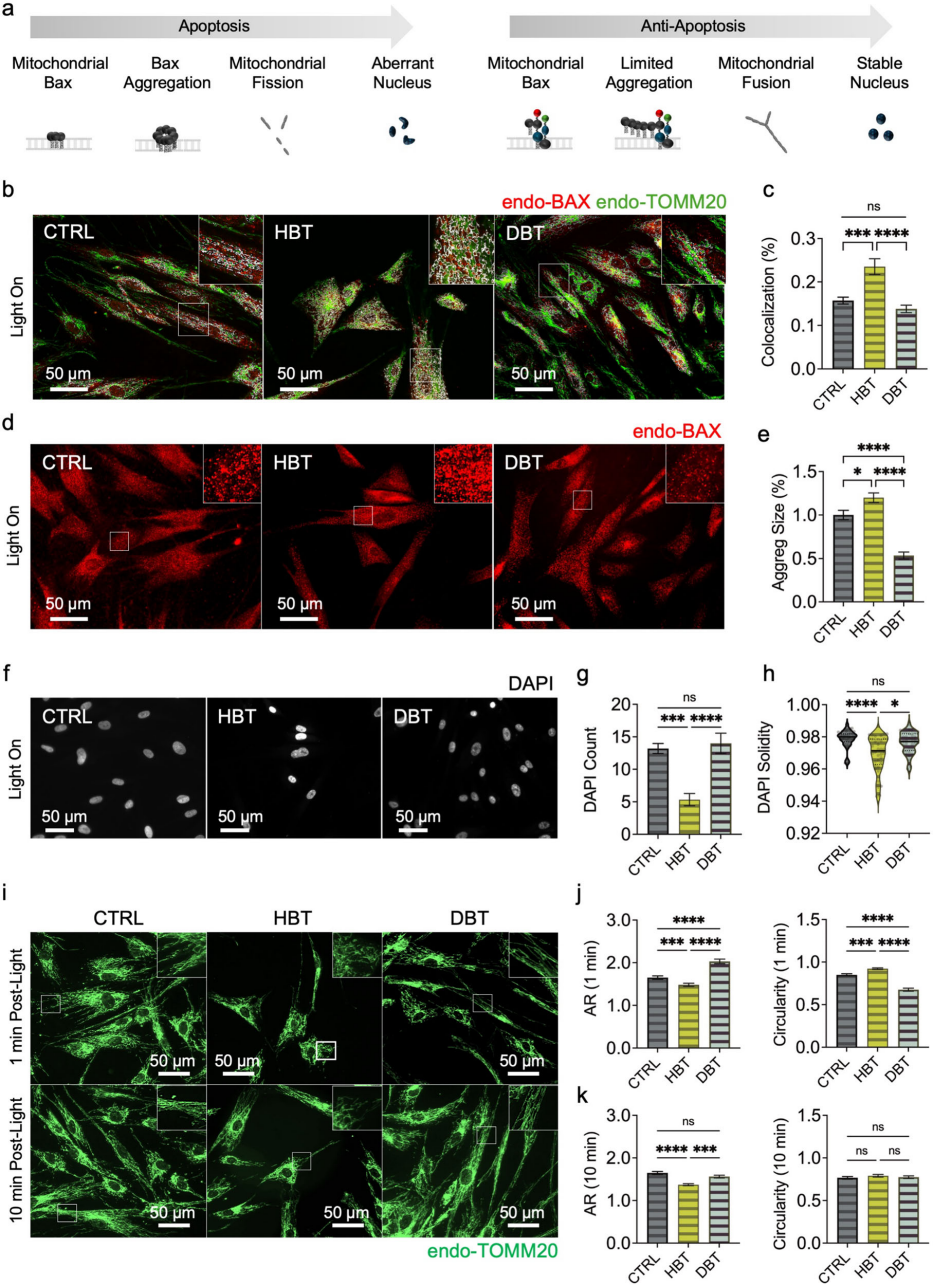
Assessment of BAX translocation to mitochondria, nuclear aberrations and mitochondrial morphology. **a** A schematic representation of sequential events in apoptosis versus antiapoptotic pathways. In the apoptotic scenario, BAX translocates to mitochondria and aggregates, triggering fission and aberrant nuclear morphology. Conversely, the DBT unit limits mitochondrial BAX aggregation, thereby preserving fusion and maintaining stable nuclear morphology. **b** Representative immunofluorescence images of endo-BAX (red) and endo- TOMM20 (green). White regions indicate BAX–mitochondria colocalization. **c** A quantification of BAX–mitochondria colocalization (n = 10 per group). Statistical analysis was performed using the Kruskal–Wallis test with Dunn’s multiple comparisons. ***P < 0.001; ****P < 0.0001. **d** Microscopic images of endo-BAX aggregation using size-filtered puncta analysis. **e** A quantification of BAX aggregate size (n = 300 per group) analyzed by the Kruskal–Wallis test with Dunn’s multiple comparisons. *P < 0.05; ****P < 0.0001; ns, non-significant. f Representative DAPI-stained nuclei. **g** A quantification of cell number based on DAPI counts per ROI (n = 10 per group) analyzed by one-way ANOVA with Tukey’s post hoc test. **h** Analysis of nuclear solidity (defined as the ratio of nuclear area to its convex hull area; n = 40 per group). Data were analyzed by the Kruskal–Wallis test with Dunn’s multiple comparisons. *P < 0.05; ***P < 0.001; ****P < 0.0001; ns, non-significant. **i** Representative images of mitochondrial morphology in cells labeled with anti-TOMM20, captured immediately after 20 min blue light irradiation or following an additional 10 min dark incubation post irradiation. **j**, **k** A qantification of mitochondrial aspect ratio (AR) and circularity (n = 300 per group) analyzed by the Kruskal–Wallis test with Dunn’s multiple comparisons. *P < 0.05; ***P < 0.001; ****P < 0.0001; ns, non-significant; Q10 mean ± s.e.m. for all data. See also Supplementary Figs. 10–16 for more details.

In this study, DBAX^S184E^ was engineered to exert controlled effects on endo-BAX. Under blue light irradiation, two distinct binding events occur: one between rBAX and rTOMM20 for locational control of rBAX, and another between rBAX and endo-BAX via the intact BH3 domain, which ultimately hinders BAX-mediated MOMP (Fig. 4a). To assess the binding pattern of DBAX^S184E^ in cells, protein aggregation events were simulated under three conditions: (1) only endo-BAX is present, (2) endo-BAX and HBAX^S184E^ are present in a 6:4 ratio (endo-HBAX) or (3) endo-BAX and DBAX^S184E^ are present in a 6:4 ratio (endo-DBAX) (Fig. 4b). The total number of proteins for simulation in each condition was set to 10. According to the simulation results, both the endo-BAX complex and the endo-HBAX complex showed pore generation, but the endo-DBAX complex hardly showed pore formation. DBAX^S184E^ was also proven to interact with endo-BAX via BH grooves. In addition, the results confirmed normal CRY2 homodimerization events^34^. To further investigate the membrane embedding pattern of the DBT unit in cells, protein aggregation events were simulated under two conditions: (1) endo-BAX, HBAX^S184E^, and rTOMM20 are present in a 5:3:2 ratio (endo-HBT), or (2) endo-BAX, DBAX^S184E^, and rTOMM20 are present in a 5:3:2 ratio (endo-DBT) (Fig. 4c). The total number of proteins for simulation in each condition was set to 10. The condition where only endo-BAX is present was also included as a control for reference. According to the simulation results, both the aggregation of endo-BAX alone and the endo-HBT condition showed deeply embedded residues (indicated in pink), whereas the endo-DBT condition showed minimally embedded residues in the complex. Furthermore, pore formation was still observed in the endo-HBT condition but not in the endo-DBT condition. These simulation results support that the genetic design of DBAX^S184E^ effectively interacts with endo-BAX, impeding pore formation by interacting with endo-BAX.

### Irradiation-driven rBAX–rTOMM20 conjugation regulates endo-BAX assembly

To verify the optogenetic controllability of the newly developed recombinant constructs, we examined the subcellular colocalization of rBAX and rTOMM20 (Fig. 2a–e). For each condition, random subcellular ROIs were selected to generate fluorescence intensity profiles, with two representative examples shown. Multiple cells were also analyzed independently to confirm reproducibility (Fig. 2f).

Under HBT conditions, blue light irradiation (‘Light On’) yielded nearly identical fluorescence intensity patterns (gray values) for rBAX and rTOMM20 across all sampled ROIs, indicative of colocalization, whereas similarity was substantially lower under ‘Light Off’ conditions (Fig. 2a, b and Supplementary Figs. 8 and 9). Notably, endo-BAX intensity also increased under Light On, suggesting that, rather than a rapid upregulation of protein expression (given the ~30-min time frame), the enhanced signal reflects the collective recruitment of endo-BAX triggered by HBT activation. Under DBT conditions, rBAX similarly exhibited a light-dependent colocalization pattern with rTOMM20 (Fig. 2c, d), although overall endo-BAX levels in DBT cells were lower than those observed under HBT Light On conditions.

Correlation analyses with simple linear regression model corroborated these observations. The correlation between rBAX and rTOMM20 intensities strengthened under blue light stimulation in both HBT Light On (HBT+, *R*² = 0.874, *P* < 0.0001) compared with HBT Light Off (HBT−, *R*² = 0.302, *P* < 0.0001) and DBT Light On cells (DBT+, *R*² = 0.852, *P* < 0.0001) compared with DBT Light Off (DBT−, *R*² = 0.118, *P* < 0.0001), with a greater disparity observed under DBT conditions (Fig. 2e). When the correlation between endo-BAX and rBAX intensities was similarly evaluated, HBT cells showed a marked increase upon light exposure (*R*² = 0.508, *P* < 0.0001) compared with HBT Light Off (*R*² = 0.197, *P* < 0.0001), consistent with HBT’s established proapoptotic function. By contrast, DBT cells exhibited similar correlations regardless of light conditions (*R*² = 0.404, *P* < 0.0001 under Light Off and *R*² = 0.419, *P* < 0.0001 under Light On).

To further quantify these findings on a per-cell level, mCherry–GFP overlap (Coloc%) was measured across multiple cells (Fig. 2f). Both HBT and DBT groups exhibited statistically significant differences between Light Off and Light On conditions (*P* < 0.0001 for both), confirming that blue light irradiation effectively induces rBAX–rTOMM20 conjugation in both constructs, albeit with distinct outcomes for endo-BAX recruitment.

Ultimately, a FRET assay was employed to demonstrate energy transfer between the two closely bound recombinant proteins under blue light irradiation (Fig. 2g). The assay was conducted in cells transfected with either HBT or DBT constructs and subjected to progressively longer blue light exposures (5 or 10 min). Irradiation time points of 5 and 10 min were analyzed, given that CRY2–CIB1 association/dark reversion operates on a minute scale and BAX exhibits biphasic membrane engagement. Specifically, the 5-min time point captures near-peak recruitment and initial BAX engagement, whereas the 10-min time point reflects ongoing oligomer maturation and stabilization^1^. Donor-only conditions were established by expressing rTOMM20 without any acceptor protein. Under these donor-only conditions, no significant FRET increase was detected following either 5 or 10 min of irradiation in cells transfected with 50 ng of plasmid per 20,000 cells (*P* = 0.9560 for 0 versus 5 min; *P* = 0.6340 for 0 versus 10 min; *P* = 0.8040 for 5 versus 10 min). Similarly, cells transfected with 200 ng of plasmid per 20,000 cells showed no significant FRET increase (*P* = 0.9610 for 0 versus 5 min; *P* = 0.6570 for 0 versus 10 min; *P* = 0.8160 for 5 versus 10 min) (Fig. 2h).

In HBT-transfected cells, 5 min of blue light exposure did not significantly elevate FRET signals (*P* = 0.9630), whereas 10 min led to a significant increase (*P* = 0.0200), and the mean difference between 5 and 10 min was also significant (*P* = 0.0382) in cells transfected with 50 ng. With 200 ng, both 5 and 10 min of irradiation induced increased FRET (*P* = 0.0402; *P* = 0.0445), with no significant mean difference (*P* = 0.9990) between these two time points.

In DBT-transfected cells, 5 min of light exposure did not produce a significant FRET increase (*P* = 0.3520), but a marked increase occurred at 10 min (*P* < 0.0001), and the mean difference between 5 and 10 min was significant (*P* = 0.0003) in cells transfected with 50 ng. When 200 ng was used, both 5 and 10 min of blue light exposure induced significant FRET increases (*P* = 0.0022; *P* = 0.0147), with no significant mean difference between the two time points (*P* = 0.7785). These data confirm that prolonged blue light irradiation promotes rBAX–rTOMM20 conjugation in HBT and DBT systems, with a more rapid and extensive response at higher plasmid concentrations.

Based on these validated optogenetic modulations of the recombinant proteins, the antiapoptotic effects of DBT were subsequently evaluated by analyzing multiple cell-death-related indices and comparing cells transfected with rTOMM20 alone (control, CTRL) or HBT against DBT-transfected cells.

### Light-activated DBTs reduce BAX mitochondrial localization and aggregation and preserve nuclear and mitochondrial integrity, enhancing cell viability

The morphological assessment of apoptosis in cells was based on three key indicators. First, translocation of BAX into the mitochondria was observed, and BAX aggregation, which increases during active apoptosis, was quantified^35,36^. Second, the number and morphology of nuclei stained with 4′,6-diamidino-2-phenylindole (DAPI) were analyzed as a key indicator of cell death^37,38^. Lastly, mitochondrial fission status, determined by measuring mitochondrial length and size, was analyzed to assess the mitochondrial integrity of the cell^35,39,40^ (Fig. 3a). These assessments were performed by analyzing immunostained images of endo-BAX and endogenous TOMM20 (endo-TOMM20) to verify the effects of recombinant protein manipulation on their endogenous counterparts (Supplementary Figs. 10–12). The results presented here focus solely on the blue light-irradiated conditions; comparisons with no-light controls are provided in the supplementary information (Supplementary Figs. 13–16).

First, the colocalization of endo-BAX with mitochondria was evaluated following blue light irradiation. This evaluation was based on the established property that endo-BAX is translocated to the MOM during active apoptosis^2^. The red and green overlapping region in the images, which indicates mitochondrial BAX, was selectively filtered (white), and its area was quantified (Fig. 3b, c). The results showed that endo-BAX recruitment to the mitochondria was significantly enhanced under the proapoptotic HBT condition (*P* = 0.0003 versus CTRL; *P* < 0.0001 versus DBT), whereas it remained attenuated under DBT, with no significant difference from CTRL (*P* = 0.5015). This indicates that rBAX in the DBT construct does not facilitate robust BAX translocation to the mitochondria, unlike the HBT construct.

To further assess BAX aggregation size, a critical index of MOMP, immunofluorescence images were processed to remove outliers such as debris and highly saturated areas (Fig. 3d). Given that BAX proteins form larger clusters through redistribution as MOMP progresses, the sizes of BAX aggregates were compared and analyzed across experimental groups^41^ (Fig. 3e). In the DBT condition, the size of BAX clusters within each ROI was markedly smaller than in both the CTRL (*P* < 0.0001) and HBT (*P* < 0.0001) conditions. Moreover, the reduction in aggregate size in DBT compared with HBT was significantly greater than that observed between CTRL and HBT (*P* = 0.0442), highlighting the pronounced attenuation of endo-BAX aggregation in the DBT condition to levels below baseline. Altogether, the mitochondrial colocalization of BAX and aggregate size measurements underscore the antiapoptotic efficacy of the DBT construct in modulating neighboring endo-BAX.

Second, cellular viability was evaluated by examining the number and morphology of nuclei via DAPI staining under blue light irradiation in CTRL, HBT and DBT conditions (Fig. 3f). As shown in Fig. 3g, quantitative analysis revealed that the DAPI counts in DBT-transfected cells were identical to those in the CTRL group (*P* = 0.8750) and significantly higher than those observed in HBT-transfected cells (*P* < 0.0001). By contrast, the HBT condition exhibited a marked reduction in nuclei number compared to CTRL (*P* = 0.0002). Furthermore, nuclear solidity, a measure of structural integrity, was well maintained in DBT-transfected cells, with values comparable to CTRL (*P* = 0.3767) and significantly superior to those in the HBT condition (*P* = 0.0213) (Fig. 3h). Notably, the HBT condition showed considerable nuclear distortion relative to CTRL (*P* < 0.0001). These results underscore that DBT not only preserves nuclear integrity but also maintains overall cellular viability under blue light irradiation.

Third, to confirm the protective effects of DBTs on mitochondrial integrity, mitochondrial morphology was examined. Guided by prior optogenetic-apoptosis kinetics showing apoptotic onset ~20 min after light initiation, we quantified mitochondrial morphology at 1 and 10 min post irradiation to capture early translocation and intermediate remodeling phases^2^. Cells irradiated with blue light under each condition were immunostained with an anti-TOMM20 antibody to visualize mitochondrial structures, which reflect either fission or fusion states (Fig. 3i). Active mitochondrial fission, commonly associated with cellular stress or damage, is characterized by shorter and more circular mitochondria that can be quantitatively assessed^40^. Mitochondrial morphology was evaluated at two time points: immediately after blue light irradiation (1 min post irradiation; Fig. 3j) and 10 min post irradiation (following 10 min of dark incubation; Fig. 3k). In samples prepared immediately post irradiation, the aspect ratio was significantly higher in the DBT condition than in CTRL (*P* < 0.0001) and HBT (*P* < 0.0001), whereas HBT exhibited a lower aspect ratio compared with CTRL (*P* = 0.0008). The index of circularity, a marker of dot-like mitochondrial fission, was also significantly elevated in the HBT condition relative to CTRL (*P* = 0.0002) but was reduced in DBT-transfected cells compared with both CTRL (*P* < 0.0001) and HBT (*P* < 0.0001) conditions.

After 10 min of dark incubation following blue light irradiation, these trends were somewhat attenuated. In the DBT condition, no significant differences were observed in either aspect ratio or circularity between DBT and CTRL (*P* = 0.4185 and *P* > 0.9999, respectively). However, the mitochondrial aspect ratio of DBT-transfected cells remained significantly higher than that in the HBT condition (*P* = 0.0001). While the aspect ratio in the HBT condition continued to be lower than that in the CTRL condition (*P* < 0.0001), circularity was restored to levels comparable to both CTRL (*P* = 0.6749) and DBT (*P* > 0.9999) during the dark incubation. This recovery may be attributed to rapid mitochondrial dynamics that promote the fusion of highly circular, fissioned mitochondria^42,43^. These results indicate that the antiapoptotic effect conferred by DBT, evidenced by sustained mitochondrial fusion, is robust enough to maintain mitochondrial morphology comparable to the baseline levels observed in the CTRL group. This effect is rapidly reversed by dark incubation, probably due to the intrinsic rapid reversibility of optogenetic systems.

### DBT preserves mitochondrial integrity and suppresses APAF1/caspase-3–dependent apoptotic signaling under apoptotic drug treatment

**Fig. 5:**
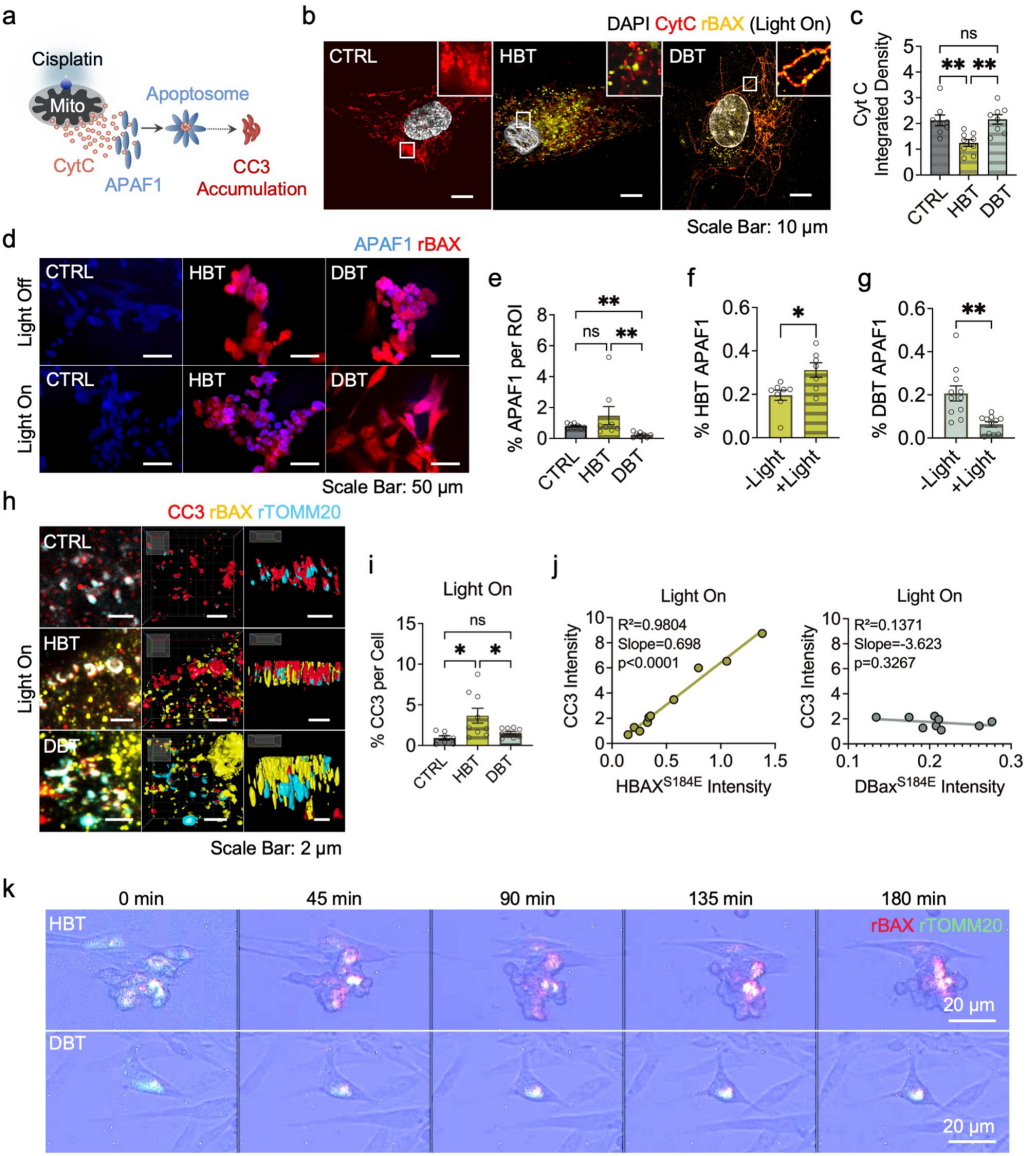
Assessment of DBT antiapoptotic functionality following cisplatin-induced apoptosis using immunofluorescence and live-cell imaging. **a** A schematic of the experimental design. The cells were treated with cisplatin to induce apoptosis, followed by 10 min of blue light irradiation. Cisplatin triggers CytC release from mitochondria into the cytosol, where it binds APAF1 to form the apoptosome, leading to the accumulation of CC3. **b** CytC localization and rBAX distribution. Immunofluorescence analysis revealed that intact cells retain CytC within mitochondria, whereas apoptotic cells exhibit a diffuse, smudged CytC pattern indicative of leakage. A decrease in CytC integrated density indicates leakage. **c** A bar graph illustrating the decrease in CytC integrated density in the HBT condition, with statistical significance determined by one-way ANOVA and Tukey’s post hoc test (*n* = 9 per group). **P < 0.01; ns, non-significant. **d** A visualization of APAF1 accumulation in each condition. **e** A quantification of APAF1 accumulation under blue light irradiation was performed using the Kruskal–Wallis test with Dunn’s multiple comparisons (*n* = 8 per group) (see also Supplementary Figs. 20 and 21 for more details). **f**, **g** rBAX–APAF1 colocalization was assessed using an unpaired *t*-test for HBT (*n* = 8) (**f**) and the Mann–Whitney test for DBT (*n* = 11) (**g**). *P < 0.05; **P < 0.01; ns, non-significant. **h**–**j** CC3 accumulation and its colocalization with the rBAX–rTOMM20 unit. **h** Representative 3D-rendered *z*-stack images illustrate CC3 colocalizing with rBAX–rTOMM20 following drug treatment and blue light irradiation. **i** Analysis of normalized CC3 levels per cell was performed using one-way ANOVA with Tukey’s post hoc test (*n* = 8 per group). **j** To evaluate rBAX–CC3 colocalization, a correlation analysis was conducted under blue light conditions, with the *R*², slope and *P* value reported (*n* = 8 per group). **k** Live-cell imaging. Time-lapse recordings over 3 h demonstrate the spatial overlap of recombinant proteins and progressive changes in cell morphology; mean ± s.e.m. for all data. See also Supplementary Figs. 17–24 and Supplementary Video 1 for more details.

Finally, the antiapoptotic effects of DBTs were assessed by exposing cells to 200 μM cisplatin for 10 min with or without irradiation, a compound known to induce MOMP^44^. Upon cisplatin treatment, mitochondrial CytC is released into the cytosol, where it binds to APAF1 to form the apoptosome, triggering downstream signaling cascades that result in the accumulation of cleaved caspase-3 (CC3) (Fig. 5a). To evaluate this process, the spatial distribution of key apoptotic markers including CytC, APAF1 and CC3 were investigated after cisplatin treatment.

First, CytC distribution was examined to assess the preservation of mitochondrial integrity (Fig. 5b, c and Supplementary Figs. 17 and 18). Immunofluorescence images revealed that apoptotic cells exhibit diffuse, smudged CytC signals indicative of leakage, alongside visible mitochondrial structures. In HBT-transfected cells, the integrated fluorescence density of CytC was significantly attenuated (*P* = 0.0048 versus CTRL; *P* < 0.0034 versus DBT), indicating a dispersed, noncontained distribution of CytC. By contrast, no significant difference was observed between DBT-transfected cells and control cells (*P* = 0.9881). Moreover, when mitochondrial integrity was evaluated by measuring the aspect ratio of CytC distribution, DBT-transfected cells demonstrated superior preservation relative to both the CTRL (*P* = 0.0391; one-way analysis of variance (ANOVA), Tukey’s post hoc test, *n* = 300 for all) and HBT conditions (*P* = 0.0093, *n* = 300), whereas no significant difference was observed between CTRL and HBT conditions (*P* = 0.8716) (Supplementary Fig. 17d). These effects were not observed under no-light control conditions (Supplementary Fig. 18b, c).

Second, APAF1 accumulation was assessed. Given that baseline APAF1 expression is broad and variable, a high dose of cisplatin (1 mM) was used to elicit a robust apoptotic response, ensuring drug-specific APAF1 accumulation for apoptosome assembly (Fig. 5d–g). Immunofluorescence microscopy revealed extensive cell death following drug treatment across all conditions, with a significant reduction observed in DBT-transfected cells under blue light irradiation. Quantitative analysis demonstrated that APAF1 intensity in DBT-transfected cells under irradiation was significantly lower than that in both CTRL (*P* = 0.0023) and HBT (*P* = 0.0027) groups (Fig. 5e). By contrast, no significant difference in APAF1 accumulation was detected between CTRL and HBT groups, probably due to the harsh drug treatment obscuring subtle differences (*P* > 0.9999). Furthermore, in DBT-transfected cells, APAF1-rBAX colocalization was markedly attenuated under blue light conditions compared to no-light conditions (*P* = 0.0014) (Fig. 5f), whereas HBT-transfected cells showed a significant increase in colocalization under blue light irradiation (*P* = 0.0130) (Fig. 5g).

Third, CC3 accumulation and its colocalization with rBAX were evaluated by immunofluorescence microscopy that highlighted the localized distribution of CC3 around mitochondria (Fig. 5h–j and Supplementary Figs. 19–21). The 3D reconstructions revealed pronounced colocalization of CC3 with the HBT complex, whereas DBT-transfected cells displayed less colocalization (Fig. 5h). Quantitative analysis of immunofluorescence images indicated that overall CC3 concentrations did not differ significantly between groups under dark (*P* = 0.0032) (Supplementary Fig. 21a, c). Under blue light irradiation, DBT-transfected cells exhibited significantly lower CC3 levels compared with HBT-transfected cells (*P* = 0.0446), highlighting DBT’s capacity to attenuate apoptotic signaling (Fig. 5i and Supplementary Fig. 20a). CC3 accumulation in HBT-transfected cells was significantly higher than in CTRL (*P* = 0.0129), whereas DBT-transfected cells remained comparable to CTRL (*P* = 0.7456).

To further elucidate the relationship between rBAX and CC3 distributions, simple linear regression analysis was performed. Under blue light conditions, HBT-transfected cells displayed a strong positive correlation between rBAX and CC3 (*R*² = 0.9804; *P* < 0.0001), whereas DBT-transfected cells showed no significant correlation (*R*² = 0.1371; *P* = 0.3267). Under no-light conditions, no significant correlations were observed in either condition (Supplementary Fig. 21b). These findings indicate that DBT-transfected cells exhibit attenuated CC3 accumulation, reinforcing the antiapoptotic efficacy of the DBT construct.

Finally, to observe the antiapoptotic function of DBTs in real time, a live-cell imaging assay was performed with 1000 ms blue light pulses every 5 min over a 3-h period under drug treatment (Fig. 5k). The results showed that HBT and DBT recombinant proteins gradually interacted over time. Notably, DBT-transfected cells retained their morphological integrity, whereas HBT-transfected cells exhibited progressive structural disruption.

Integrating these observations with data obtained across a range of apoptotic inducer experiments highlights the robust antiapoptotic functionality of DBTs (Supplementary Figs. 22–24). This protective effect is attributed to the unique structural features of DBTs, which deter pore formation. Collectively, these findings highlight the potential of the structural modifications proposed in this study to modulate BAX activity and promote antiapoptotic responses, offering promising prospects for future drug development.

## Discussion

Recognizing the importance of modulating apoptosis in cells, this study proposes BAX as a promising therapeutic target for apoptosis-associated diseases. This potential is attributed not only to its organelle-specific assembly but also to its intrinsic interaction mechanism, whereby a single dysfunctional BAX molecule can influence neighboring BAX proteins, thereby altering the dynamics of apoptotic signaling. In our work, we employed an optogenetic approach to direct BAX to the mitochondria, the primary site of its apoptotic activity, and to elicit antiapoptotic effects within BAX assemblies. Consequently, apoptosis was attenuated through targeted alterations that prevented pore formation in the MOM. This system was activated simply by exposure to blue light.

Mitochondria, which serve as the primary platform for BAX function, undergo dynamic cycles of fission, fusion, and transport on timescales ranging from seconds to hours, thus playing a vital role in cellular quality control. Under certain stimuli or stress conditions, they can rapidly eliminate damaged components or quickly compensate for functional deficits, enabling swift adaptation^39,40,45^. Typically, upon receiving cell death signals, mitochondria undergo pronounced fission, prompting CytC release and accelerating proapoptotic processes, thus mitochondrial dynamics are important in regulating cell death^6^. During this process, multiple BAX proteins respond to apoptotic cues by undergoing structural rearrangements and integrating into the MOM within minutes, where they form pores that compromise mitochondrial integrity^3,4^. BAX is also thought to interact directly with mitochondrial fission mediators such as DRP1, thereby exerting a substantial influence on mitochondrial morphology^46,47^.

Accordingly, we aimed to regulate BAX’s mitochondrial binding event by hindering the interaction of its hydrophobic motif, which serves as a mitochondrial anchor. To achieve this, we engineered an optogenetic system that strategically positions CRY2 over the hydrophobic motif, thereby promoting its binding to CIB1-fused TOMM20, a mitochondrial membrane protein and effectively impeding BAX-mediated pore formation. Moreover, this design repositions the BH groove away from the MOM, further disrupting collective BAX interactions (Figs. 1 and 2).

In our study, we confirmed that CRY2 and CIB1 bind tightly under blue light and exhibit more rapid interactions at higher expression levels in cells (Figs. 2 and 3). This light-induced interaction drove the translocation of rBAX to the mitochondria and influenced the recruitment of endo-BAX (Fig. 4).

Throughout this study, we compared HBT, a system previously demonstrated to be reliable and that preserves the native BAX structure, with DBT, as well as with a rTOMM20-only condition (CTRL) that exhibits stable transient expression of recombinant proteins without facilitating apoptosis^28^. Our findings reveal that DBT not only diminishes the mitochondrial localization of endo-BAX but also suppresses its aggregation (Figs. 3c, d and 4c–e). With respect to localization, DBT shows transient mitochondrial association followed by dissociation, suggesting perturbation of steady-state BAX aggregation on MOM (Fig. 1 and Supplementary Fig. 5). Accordingly, DBT-expressing cells exhibited fewer endo-BAX aggregates than controls, consistent with a model in which DBT engages the BH3 interface for BAX interaction, reduces the stable oligomerization of endo-BAX at the MOM. By contrast, HBT displays stronger and more persistent membrane anchoring, which facilitates the recruitment and oligomerization of endo-BAX (Fig. 4a, b, e). As a result, DBT mitigates cell death, enhances cell viability, and promotes sustained mitochondrial fusion (Fig. 4f–k). In essence, DBT functions as a covert modulator among BAX molecules, effectively inactivating their proapoptotic activity and reducing apoptosis to levels below those observed in control cells.

We further conducted a comparative analysis of HBT and DBT under drug-induced stress by exposing cells to high concentrations of cisplatin, followed by light illumination (Fig. 5 and Supplementary Figs. 17–24). Under these conditions, we tested mitochondrial CytC release, APAF1 aggregation and subsequent CC3 accumulation (Fig. 5 and Supplementary Figs. 19–21). Overall, the DBT construct markedly mitigated these effects. The maintenance of CytC distribution and mitochondrial integrity in DBT-transfected cells, as evidenced by integrated fluorescence density and aspect ratio measurements, suggests that DBT effectively preserves mitochondrial function under stress (Fig. 5b-c and Supplementary Fig. 17). Moreover, the reduced APAF1 accumulation and diminished CC3 levels in DBT-transfected cells, coupled with the lack of significant rBAX–CC3 correlation, indicate that DBT not only disrupts BAX’s ability to promote apoptotic pore formation but also attenuates downstream apoptotic signaling (Fig. 5d–j). The background studies show that APAF1 rapidly redistributes to mitochondrial subdomains and assembles the apoptosome immediately downstream of MOMP^31^. Accordingly, these results confirm that APAF1 function is weakened under DBT condition.

Live-cell imaging further confirmed these findings by demonstrating sustained cellular integrity over time in DBT-transfected cells, in contrast to the progressive morphological deterioration observed in HBT-transfected cells (Fig. 5k). Collectively, these results support the hypothesis that the novel optogenetic modification of BAX structure can effectively suppress its proapoptotic activity, thereby offering a promising strategy for modulating apoptosis in therapeutic contexts.

Even though this novel technology offers great future potential for addressing disease-related challenges, there remain limitations that must be overcome to enhance its utility. One limitation of this study is the inherent toxicity associated with transfection reagents, which complicates the accurate evaluation of recombinant proteins in apoptosis-related research. Future studies should consider integrating advanced transfection methodologies to remove these effects and enable more precise assessments. In addition, further validation across a broader range of cell types is necessary, with particular emphasis on neuronal cells, where preventing unwarranted apoptosis is critical. It is also important to note that inhibiting cell death does not inherently equate to improved health. Indeed, excessive suppression of apoptosis may lead to inefficient energy utilization, accelerated aging, or compromised quality control mechanisms. Therefore, careful deliberation is required in the development and application of this technology. Another potential limitation of this system is light-induced CRY2 homo-oligomerization at high expression or illumination levels and phototoxicity from blue light exposure, although both can be minimized through optimized expression and irradiation conditions^4,5^. Although we did not directly evaluate CRY2 homo-oligomerization in this study, further investigation will be essential to enable future refinement and broader application of the system. Moreover, the simulation modeling was constrained by capacity limit, so outputs should be viewed as a comparative framework and require further experimental validation. Lastly, a comprehensive evaluation of the interplay between apoptosis and regeneration is indispensable, as the therapeutic potential of such interventions is likely to be maximized when these processes are balanced.

Further studies will be needed to fully elucidate this model’s mechanism. In particular, regarding the interaction of BAX with DRP1 via the N-terminus, our constructs that modify this region probably exert opposite effects. DBT’s transient membrane residency may weaken sustained DRP1 recruitment and thereby reduce mitochondrial fission, whereas HBT’s stable anchoring may enhance DRP1 engagement and promote fission^6,7^. Development of other apoptotic mediators as optogenetic functional modules should also be considered, since many proteins, such as BAK, share similar mechanisms of being anchored or embedded in the MOM to mediate MOMP and apoptosis^8^. For in vivo application of our constructs, several important factors should be taken into account. Minimizing functional domains and retaining only the hydrophobic-binding regions of TOMM20 may reduce the risk of mitochondrial functional disturbance or overexpression artifacts. For the HBT and DBT constructs, our in vitro data indicate that autoactivation-related toxicity occurs only when the BAX 184 sequence is intact, whereas the S184E mutation prevents cell death under dark conditions in fibroblasts (Supplementary Fig. 7). However, this must be carefully evaluated with organ- and dose-specific optimization. Although we did not observe off-target effects beyond light-triggered activity in cultured cells, systemic delivery in vivo may pose additional challenges, including tissue tropism and immune recognition. Therefore, rigorous evaluation of acute and chronic toxicity, immune responses and off-target activation in appropriate animal models will be essential to ensure biosafety for translational applications.

Given the prevalence of age-related diseases associated with apoptosis, including dementia, Parkinson’s disease, arthritis and diabetes, this study elucidates critical mechanisms for precisely targeting BAX by examining how a single genetically engineered variant, designed to hinder its mitochondrial permeabilization, disrupts the cell’s ability to mediate and trigger apoptosis. By overcoming several of the noted limitations, we expect that our results will establish a solid foundation for evaluating antiapoptotic interventions in cells and facilitate the advancement of novel therapies designed to reduce apoptosis in forthcoming disease treatments.

## Supplementary information

Supplementary Information

Comparison of apoptosis under drug conditions in HBT- and DBT-transfected cells.

## Data Availability

Data are available upon request.

## References

[1] Cory, S. & Adams, J. M. The Bcl2 family: regulators of the cellular life-or-death switch. *Nat. Rev. Cancer* **2**, 647–656 (2002). 12209154 10.1038/nrc883

[2] Cosentino, K. & García-Sáez, A. J. Bax and Bak pores: are we closing the circle?. *Trends Cell Biol.* **27**, 266–275 (2017). 27932064 10.1016/j.tcb.2016.11.004 PMC5898608

[3] Gavathiotis, E., Reyna, D. E., Davis, M. L., Bird, G. H. & Walensky, L. D. BH3-triggered structural reorganization drives the activation of proapoptotic BAX. *Mol. Cell* **40**, 481–492 (2010). 21070973 10.1016/j.molcel.2010.10.019 PMC3050027

[4] Annis, M. G. et al. Bax forms multispanning monomers that oligomerize to permeabilize membranes during apoptosis. *EMBO J.* **24**, 2096–2103 (2005). 15920484 10.1038/sj.emboj.7600675 PMC1150878

[5] Suzuki, M., Youle, R. J. & Tjandra, N. Structure of Bax. *Cell* **103**, 645–654 (2000). 11106734 10.1016/s0092-8674(00)00167-7

[6] Peña-Blanco, A. & García-Sáez, A. J. Bax, Bak and beyond—mitochondrial performance in apoptosis. *FEBS J.* **285**, 416–431 (2018). 28755482 10.1111/febs.14186

[7] Bagci, E. Z., Vodovotz, Y., Billiar, T. R., Ermentrout, G. B. & Bahar, I. Bistability in apoptosis: roles of Bax, Bcl-2, and mitochondrial permeability transition pores. *Biophys J.* **90**, 1546–1559 (2006). 16339882 10.1529/biophysj.105.068122 PMC1367306

[8] Tan, C. et al. Auto-activation of the apoptosis protein Bax increases mitochondrial membrane permeability and is inhibited by Bcl-2. *J. Biol. Chem.* **281**, 14764–14775 (2006). 16571718 10.1074/jbc.M602374200 PMC2826894

[9] Teles, A. V. F. F. et al. Increase in bax expression and apoptosis are associated in Huntington’s disease progression. *Neurosci. Lett.* **438**, 59–63 (2008). 18468793 10.1016/j.neulet.2008.03.062

[10] Yin, C., Knudson, C. M., Korsmeyer, S. J. & Dyke, T. Van. Bax suppresses tumorigenesis and stimulates apoptosis in vivo. *Nature* **385**, 637–640 (1997). 9024662 10.1038/385637a0

[11] MacGibbon, G. A. et al. Bax expression in mammalian neurons undergoing apoptosis, and in Alzheimer’s disease hippocampus. *Brain Res.* **750**, 223–234 (1997). 9098548 10.1016/s0006-8993(96)01351-0

[12] Aggarwal, S. & Gupta, S. Increased apoptosis of T cell subsets in aging humans: altered expression of Fas (CD95), Fas ligand, Bcl-2, and Bax. *J. Immunol.* **160**, 1627–1637 (1998). 9469419

[13] Takeuchi, O. et al. Essential role of BAX,BAK in B cell homeostasis and prevention of autoimmune disease. *Proc. Natl Acad. Sci. USA* **102**, 11272–11277 (2005). 16055554 10.1073/pnas.0504783102 PMC1183578

[14] Lee, Y. & Gustafsson, ÅB. Role of apoptosis in cardiovascular disease. *Apoptosis* **14**, 536–548 (2009). 19142731 10.1007/s10495-008-0302-x

[15] Kim, N.-H. & Kang, P. M. Apoptosis in cardiovascular diseases: mechanism and clinical implications. *Korean Circ. J.* **40**, 299 (2010). 20664736 10.4070/kcj.2010.40.7.299 PMC2910284

[16] Carneiro, B. A. & El-Deiry, W. S. Targeting apoptosis in cancer therapy. *Nat. Rev. Clin. Oncol.* **17**, 395–417 (2020). 32203277 10.1038/s41571-020-0341-y PMC8211386

[17] Liu, Z. et al. Direct activation of bax protein for cancer therapy. *Med. Res. Rev.* **36**, 313–341 (2016). 26395559 10.1002/med.21379 PMC4752390

[18] Zhang, L., Yu, J., Park, B. H., Kinzler, K. W. & Vogelstein, B. Role of *BAX* in the apoptotic response to anticancer agents. *Science* **290**, 989–992 (2000). 11062132 10.1126/science.290.5493.989

[19] Jenner, P. & Warren Olanow, C. Understanding cell death in Parkinson's disease. *Ann. Neurol* **44**, S72–S84 (1998). 9749577 10.1002/ana.410440712

[20] Gorman, A. M. Neuronal cell death in neurodegenerative diseases: recurring themes around protein handling. *J. Cell Mol. Med.* **12**, 2263–2280 (2008). 18624755 10.1111/j.1582-4934.2008.00402.x PMC4514105

[21] Moujalled, D., Strasser, A. & Liddell, J. R. Molecular mechanisms of cell death in neurological diseases. *Cell Death Differ.* **28**, 2029–2044 (2021). 34099897 10.1038/s41418-021-00814-y PMC8257776

[22] Emiliani, V. et al. Optogenetics for light control of biological systems. *Nat. Rev. Methods Primers* **2**, 55 (2022). 37933248 10.1038/s43586-022-00136-4 PMC10627578

[23] Duan, L. et al. Understanding CRY2 interactions for optical control of intracellular signaling. *Nat. Commun.* **8**, 547 (2017). 28916751 10.1038/s41467-017-00648-8 PMC5601944

[24] Che, D. L., Duan, L., Zhang, K. & Cui, B. The dual characteristics of light-induced cryptochrome 2, homo-oligomerization and heterodimerization, for optogenetic manipulation in mammalian cells. *ACS Synth. Biol.* **4**, 1124–1135 (2015). 25985220 10.1021/acssynbio.5b00048 PMC5061508

[25] Godwin, W. C., Hoffmann, G. F., Gray, T. J. & Hughes, R. M. Imaging of morphological and biochemical hallmarks of apoptosis with optimized optogenetic tools. *J. Biol. Chem.* **294**, 16918–16929 (2019). 31582560 10.1074/jbc.RA119.009141 PMC6851291

[26] Gavathiotis, E. et al. BAX activation is initiated at a novel interaction site. *Nature* **455**, 1076–1081 (2008). 18948948 10.1038/nature07396 PMC2597110

[27] Garner, T. P. et al. An autoinhibited dimeric form of BAX regulates the BAX activation pathway. *Mol. Cell* **63**, 485–497 (2016). 27425408 10.1016/j.molcel.2016.06.010 PMC4975667

[28] Hughes, R. M. et al. Optogenetic apoptosis: light-triggered cell death. *Angewandte Chemie Int. Ed.* **54**, 12064–12068 (2015). 10.1002/anie.201506346 PMC4819321 26418181

[29] Mirdita, M. et al. ColabFold: making protein folding accessible to all. *Nat Methods* **19**, 679–682 (2022). 35637307 10.1038/s41592-022-01488-1 PMC9184281

[30] Jumper, J. et al. Highly accurate protein structure prediction with AlphaFold. *Nature* **596**, 583–589 (2021). 34265844 10.1038/s41586-021-03819-2 PMC8371605

[31] Baliga, B. & Kumar, S. Apaf-1/cytochrome c apoptosome: an essential initiatorof caspase activation or just a sideshow?. *Cell Death Differ.* **10**, 16–18 (2003). 12655291 10.1038/sj.cdd.4401166

[32] Youle, R. J. & Karbowski, M. Mitochondrial fission in apoptosis. *Nat. Rev. Mol. Cell Biol.* **6**, 657–663 (2005). 16025099 10.1038/nrm1697

[33] Doonan, F. & Cotter, T. G. Morphological assessment of apoptosis. *Methods* **44**, 200–204 (2008). 18314050 10.1016/j.ymeth.2007.11.006

[34] Ma, L. et al. Structural insights into the photoactivation of *Arabidopsis* CRY2. *Nat. Plants* **6**, 1432–1438 (2020). 33199893 10.1038/s41477-020-00800-1

[35] McArthur, K. et al. BAK/BAX macropores facilitate mitochondrial herniation and mtDNA efflux during apoptosis. *Science* **359**, eaao6047 (2018). 29472455 10.1126/science.aao6047

[36] Hauseman, Z. J. et al. Homogeneous oligomers of pro-apoptotic BAX reveal structural determinants of mitochondrial membrane permeabilization. *Mol. Cell* **79**, 68–83.e7 (2020). 32533918 10.1016/j.molcel.2020.05.029 PMC7472837

[37] Nascimento, L. do, Nicoletti, N. F., Peletti-Figueiró, M., Marinowic, D. & Falavigna, A. Hyaluronic acid in vitro response: viability and proliferation profile of human chondrocytes in 3D-based culture. *Cartilage* **13**, 1077S–1087S (2021). 34775798 10.1177/19476035211057244 PMC8804839

[38] Shamseldin, H. E. et al. NUP214 deficiency causes severe encephalopathy and microcephaly in humans. *Hum. Genet.* **138**, 221–229 (2019). 30758658 10.1007/s00439-019-01979-w

[39] Garcia, D. A., Powers, A. F., Bell, T. A., Guo, S. & Aghajan, M. Antisense oligonucleotide-mediated silencing of mitochondrial fusion and fission factors modulates mitochondrial dynamics and rescues mitochondrial dysfunction. *Nucleic Acid Ther.* **32**, 51–65 (2022). 34698563 10.1089/nat.2021.0029 PMC8817704

[40] Westrate, L. M., Drocco, J. A., Martin, K. R., Hlavacek, W. S. & MacKeigan, J. P. Mitochondrial morphological features are associated with fission and fusion events. *PLoS ONE* **9**, e95265 (2014). 24733410 10.1371/journal.pone.0095265 PMC3986258

[41] Zhou, L. & Chang, D. C. Dynamics and structure of the Bax–Bak complex responsible for releasing mitochondrial proteins during apoptosis. *J. Cell Sci.* **121**, 2186–2196 (2008). 18544634 10.1242/jcs.024703

[42] Nunnari, J. et al. Mitochondrial transmission during mating in *Saccharomyces cerevisiae* is determined by mitochondrial fusion and fission and the intramitochondrial segregation of mitochondrial DNA. *Mol. Biol. Cell* **8**, 1233–1242 (1997). 9243504 10.1091/mbc.8.7.1233 PMC276149

[43] Al Ojaimi, M., Salah, A. & El-Hattab, A. Mitochondrial fission and fusion: molecular mechanisms, biological functions, and related disorders. *Membranes* **12**, 893 (2022). 36135912 10.3390/membranes12090893 PMC9502208

[44] Fang, C. et al. Natural products: potential treatments for cisplatin-induced nephrotoxicity. *Acta Pharmacol. Sin.* **42**, 1951–1969 (2021). 33750909 10.1038/s41401-021-00620-9 PMC8633358

[45] Obashi, K. & Okabe, S. Regulation of mitochondrial dynamics and distribution by synapse position and neuronal activity in the axon. *Eur. J. Neurosci.* **38**, 2350–2363 (2013). 23725294 10.1111/ejn.12263

[46] Karbowski, M. et al. Spatial and temporal association of Bax with mitochondrial fission sites, DRP1, and Mfn2 during apoptosis. *J Cell Biol* **159**, 931–938 (2002). 12499352 10.1083/jcb.200209124 PMC2173996

[47] Montessuit, S. et al. Membrane remodeling induced by the dynamin-related protein DRP1 stimulates Bax oligomerization. *Cell* **142**, 889–901 (2010). 20850011 10.1016/j.cell.2010.08.017 PMC4115189

