# Supplementary material for: Optogenetic engineering of BAX to control mitochondrial permeabilization and attenuate apoptosis in cells
Source: Exp Mol Med. 2025 Dec 26;57(12):2972–84. doi: 10.1038/s12276-025-01605-y (PMC12800262; doi:10.1038/s12276-025-01605-y)

Optogenetic Engineering of BAX to Control Mitochondrial Permeabilization and Attenuate Apoptosis in Cells

Dain Lee[1,9], Hyunjun Bae[2,3], Dongwoo Oh[1], Minseop Kim[1], Ju-Hee Kim[1], Jinchul Ahn[2,4], Seok-Hyeon Kang[2], Seo-Hee You[2], Dong-Hwee Kim[1,5,6], Hyun Jeong Oh[2], Won Do Heo[7], Seok Chung[1,2,8*]

[1]KU-KIST Graduate School of Converging Science and Technology, Korea University, Seoul 02841, Republic of Korea

[2]School of Mechanical Engineering, Korea University, Seoul 02841, Republic of Korea

[3]Department of Mechanical Engineering, Stanford University, Stanford, CA 94305, USA

[4]Center for Advanced Biomolecular Recognition, Korea Institute of Science and Technology (KIST), Seoul 02792, Republic of Korea

[5]Department of Integrative Energy Engineering, College of Engineering, Korea University, Seoul, 02841, Republic of Korea

[6]Biomaterials Research Center, Biomedical Research Division, Korea Institute of Science and Technology (KIST), Seoul 02792, Republic of Korea

[7]Department of Biological Sciences, Korea Advanced Institute of Science and Technology (KAIST), Daejeon 34141, Republic of Korea

[8]Center for Brain Technology, Brain Science Institute, Korea Institute of Science and Technology (KIST), Seoul 02792, Republic of Korea

[9]Denotes the first author.

*Correspondence: sidchung@korea.ac.kr (S.C.)

Keywords: Optogenetics, BAX, Anti-apoptosis, Cryptochrome, Mitochondria, MOMP

Supplementary Information

Supplementary Figure 1. Graphic representation of the sequences

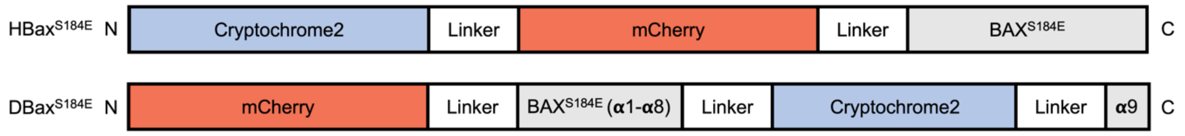

Supplementary Table 1. Gene sequences

HBT^{S184E} (CRY2::mCh::BAX^{S184E})
ATGAAGATGGACAAAAAGACTATAGTTTGGTTTAGAAGAGACCTAAGGATTGAGGATAATCCT
GCATTAGCAGCAGCTGCTCACGAAGGATCTGTTTTTCCTGTCTTCATTTGGTGTCCTGAAGAA
GAAGGACAGTTTTATCCTGGAAGAGCTTCAAGATGGTGGATGAAACAATCACTTGCTCACTTA
TCTCAATCCTTGAAGGCTCTTGGATCTGACCTCACTTTAATCAAAACCCACAACACGATTTCA
GCGATCTTGGATTGTATCCGCGTTACCGGTGCTACAAAAGTCGTCTTTAACCACCTCTATGAT
CCTGTTTCGTTAGTTCGGGACCATACCGTAAAGGAGAAGCTGGTGGAACGTGGGATCTCTGT
GCAAAGCTACAATGGAGATCTATTGTATGAACCGTGGGAGATATACTGCGAAAAGGGCAAAC
CTTTTACGAGTTTCAATTCTTACTGGAAGAAATGCTTAGATATGTCGATTGAATCCGTTATGCT
TCCTCCTCCTTGGCGGTTGATGCCAATAACTGCAGCGGCTGAAGCGATTTGGGCGTGTTCGA
TTGAAGAACTAGGGCTGGAGAATGAGGCCGAGAAACCGAGCAATGCGTTGTTAACTAGAGCT
TGGTCTCCAGGATGGAGCAATGCTGATAAGTTACTAAATGAGTTCATCGAGAAGCAGTTGATA
GATTATGCAAAGAACAGCAAGAAAGTTGTTGGGAATTCTACTTCACTACTTTCTCCGTATCTC
CATTTCGGGGAAATAAGCGTCAGACACGTTTTCCAGTGTGCCCGGATGAAACAAATTATATG
GGCAAGAGATAAGAACAGTGAAGGAGAAGAAAGTGCAGATCTTTTTCTTAGGGGAATCGGTT
TAAGAGAGTATTCTCGGTATATATGTTTCAACTTCCCGTTTACTCACGAGCAATCGTTGTTGAG
TCATCTTCGGTTTTTCCCTTGGGATGCTGATGTTGATAAGTTCAAGGCCTGGAGACAAGGCA
GGACCGGTTATCCGTTGGTGGATGCCGGAATGAGAGAGCTTTGGGCTACCGGATGGATGCA
TAACAGAATAAGAGTGATTGTTTCAAGCTTTGCTGTGAAGTTTCTTCTCCTTCCATGGAAATGG
GGAATGAAGTATTTCTGGGATACACTTTTGGATGCTGATTTGGAATGTGACATCCTTGGCTGG
CAGTATATCTCTGGGAGTATCCCCGATGGCCACGAGCTTGATCGCTTGGACAATCCCGCGTT
ACAAGGCGCCAAATATGACCCAGAAGGTGAGTACATAAGGCAATGGCTTCCCGAGCTTGCGA
GATTGCCAACTGAATGGATCCATCATCCATGGGACGCTCCTTTAACCGTACTCAAAGCTTCTG
GTGTGGAACTCGGAACAAACTATGCGAAACCCATTGTAGACATCGACACAGCTCGTGAGCTA
CTAGCTAAAGCTATTTCAAGAACCCGTGAAGCACAGATCATGATCGGAGCAGCATCTGGTGG
CGGAGGCTCGGGCGGAGGTGGGTCGGGTGGCGGCGGATCAGTCGACATGGTGAGCAAGG
GCGAGGAGGATAACATGGCCATCATCAAGGAGTTCATGCGCTTCAAGGTGCACATGGAGGG
CTCCGTGAACGGCCACGAGTTCGAGATCGAGGGCGAGGGCGAGGGCCGCCCCTACGAGGG
CACCCAGACCGCCAAGCTGAAGGTGACCAAGGGTGGCCCCCTGCCCTTCGCCTGGGACATC
CTGTCCCCTCAGTTCATGTACGGCTCCAAGGCCTACGTGAAGCACCCCGCCGACATCCCCG
ACTACTTGAAGCTGTCCTTCCCCGAGGGCTTCAAGTGGGAGCGCGTGATGAACTTCGAGGA
CGGCGGCGTGGTGACCGTGACCCAGGACTCCTCCCTGCAGGACGGCGAGTTCATCTACAAG
GTGAAGCTGCGCGGCACCAACTTCCCCTCCGACGGCCCCGTAATGCAGAAGAAGACCATGG
GCTGGGAGGCCTCCTCCGAGCGGATGTACCCCGAGGACGGCGCCCTGAAGGGCGAGATCA
AGCAGAGGCTGAAGCTGAAGGACGGCGGCCACTACGACGCTGAGGTCAAGACCACCTACAA
GGCCAAGAAGCCCGTGCAGCTGCCCGGCGCCTACAACGTCAACATCAAGTTGGACATCACC
TCCCACAACGAGGACTACACCATCGTGGAACAGTACGAACGCGCCGAGGGCCGCCACTCCA
CCGGCGGCATGGACGAGCTGTACAAGGGCAGCGGCAGCGGCAGCGGTACCATGGACGGGT
CCGGGGAGCAGCCCAGAGGCGGGGGGCCCACCAGCTCTGAGCAGATCATGAAGACAGGGG
CCCTTTTGCTTCAGGGTTTCATCCAGGATCGAGCAGGGCGAATGGGGGGGGAGGCACCCGA
GCTGGCCCTGGACCCGGTGCCTCAGGATGCGTCCACCAAGAAGCTGAGCGAGTGTCTCAAG
CGCATCGGGGACGAACTGGACAGTAACATGGAGCTGCAGAGGATGATTGCCGCCGTGGACA
CAGACTCCCCCCGAGAGGTCTTTTTCCGAGTGGCAGCTGACATGTTTTCTGACGGCAACTTC
AACTGGGGCCGGGTTGTCGCCCTTTTCTACTTTGCCAGCAAACTGGTGCTCAAGGCCCTGTG
CACCAAGGTGCCGGAACTGATCAGAACCATCATGGGCTGGACATTGGACTTCCTCCGGGAG
CGGCTGTTGGGCTGGATCCAAGACCAGGGTGGTTGGGACGGCCTCCTCTCCTACTTTGGGA

CGCCCACGTGGCAGACCGTGACCATCTTTGTGGCGGGAGTGCTCACCGCCGAGCTCACCAT
CTGGAAGAAGATGGGCTGA

DBT^{S184E} (mCh::BAX α1-α8::CRY2::α9^{S184E})

ATGGTGAGCAAGGGCGAGGAGGATAACATGGCCATCATCAAGGAGTTCATGCGCTTCAAGG
TGCACATGGAGGGCTCCGTGAACGGCCACGAGTTCGAGATCGAGGGCGAGGGCGAGGGCC
GCCCCTACGAGGGCACCCAGACCGCCAAGCTGAAGGTGACCAAGGGTGGCCCCCTGCCCT
TCGCCTGGGACATCCTGTCCCCTCAGTTCATGTACGGCTCCAAGGCCTACGTGAAGCACCCC
GCCGACATCCCCGACTACTTGAAGCTGTCCTTCCCCGAGGGCTTCAAGTGGGAGCGCGTGA
TGAACTTCGAGGACGGCGGCGTGGTGACCGTGACCCAGGACTCCTCCCTGCAGGACGGCG
AGTTCATCTACAAGGTGAAGCTGCGCGGCACCAACTTCCCCTCCGACGGCCCCGTAATGCA
GAAGAAGACCATGGGCTGGGAGGCCTCCTCCGAGCGGATGTACCCCGAGGACGGCGCCCT
GAAGGGCGAGATCAAGCAGAGGCTGAAGCTGAAGGACGGCGGCCACTACGACGCTGAGGT
CAAGACCACCTACAAGGCCAAGAAGCCCGTGCAGCTGCCCGGCGCCTACAACGTCAACATC
AAGTTGGACATCACCTCCCACAACGAGGACTACACCATCGTGGAACAGTACGAACGCGCCG
AGGGCCGCCACTCCACCGGCGGCATGGACGAGCTGTACAAGGGCAGCGGCAGCGGCAGCC
TCGAGATGGACGGGTCCGGGGAGCAGCCCAGAGGCGGGGGGCCCACCAGCTCTGAGCAG
ATCATGAAGACAGGGGCCCTTTTGCTTCAGGGTTTCATCCAGGATCGAGCAGGGCGAATGG
GGGGGGAGGCACCCGAGCTGGCCCTGGACCCGGTGCCTCAGGATGCGTCCACCAAGAAGC
TGAGCGAGTGTCTCAAGCGCATCGGGGACGAACTGGACAGTAACATGGAGCTGCAGAGGAT
GATTGCCGCCGTGGACACAGACTCCCCCCGAGAGGTCTTTTTCCGAGTGGCAGCTGACATG
TTTTCTGACGGCAACTTCAACTGGGGCCGGGTTGTCGCCCTTTTCTACTTTGCCAGCAAACT
GGTGCTCAAGGCCCTGTGCACCAAGGTGCCGGAACTGATCAGAACCATCATGGGCTGGACA
TTGGACTTCCTCCGGGAGCGGCTGTTGGGCTGGATCCAAGACCAGGGTGGTTGGGACGGC
CTCCTCTCCTACTTTGGGACGCCCACGTGGCAGGGCAGCGGCAGCGGCAGCGGTACCATGA
AGATGGACAAAAAGACTATAGTTTGGTTTAGAAGAGACCTAAGGATTGAGGATAATCCTGCAT
TAGCAGCAGCTGCTCACGAAGGATCTGTTTTTCCTGTCTTCATTTGGTGTCCTGAAGAAGAAG
GACAGTTTTATCCTGGAAGAGCTTCAAGATGGTGGATGAAACAATCACTTGCTCACTTATCTC
AATCCTTGAAGGCTCTTGGATCTGACCTCACTTTAATCAAAACCCACAACACGATTTCAGCGA
TCTTGGATTGTATCCGCGTTACCGGTGCTACAAAAGTCGTCTTTAACCACCTCTATGATCCTG
TTTCGTTAGTTCGGGACCATACCGTAAAGGAGAAGCTGGTGGAACGTGGGATCTCTGTGCAA
AGCTACAATGGAGATCTATTGTATGAACCGTGGGAGATATACTGCGAAAAGGGCAAACCTTTT
ACGAGTTTCAATTCTTACTGGAAGAAATGCTTAGATATGTCGATTGAATCCGTTATGCTTCCTC
CTCCTTGGCGGTTGATGCCAATAACTGCAGCGGCTGAAGCGATTTGGGCGTGTTCGATTGAA
GAACTAGGGCTGGAGAATGAGGCCGAGAAACCGAGCAATGCGTTGTTAACTAGAGCTTGGT
CTCCAGGATGGAGCAATGCTGATAAGTTACTAAATGAGTTCATCGAGAAGCAGTTGATAGATT
ATGCAAAGAACAGCAAGAAAGTTGTTGGGAATTCTACTTCACTACTTTCTCCGTATCTCCATTT
CGGGGAAATAAGCGTCAGACACGTTTTCCAGTGTGCCCGGATGAAACAAATTATATGGGCAA
GAGATAAGAACAGTGAAGGAGAAGAAAGTGCAGATCTTTTTCTTAGGGGAATCGGTTTAAGA
GAGTATTCTCGGTATATATGTTTCAACTTCCCGTTTACTCACGAGCAATCGTTGTTGAGTCATC
TTCGGTTTTTCCCTTGGGATGCTGATGTTGATAAGTTCAAGGCCTGGAGACAAGGCAGGACC
GGTTATCCGTTGGTGGATGCCGGAATGAGAGAGCTTTGGGCTACCGGATGGATGCATAACA
GAATAAGAGTGATTGTTTCAAGCTTTGCTGTGAAGTTTCTTCTCCTTCCATGGAAATGGGGAA
TGAAGTATTTCTGGGATACACTTTTGGATGCTGATTTGGAATGTGACATCCTTGGCTGGCAGT
ATATCTCTGGGAGTATCCCCGATGGCCACGAGCTTGATCGCTTGGACAATCCCGCGTTACAA
GGCGCCAAATATGACCCAGAAGGTGAGTACATAAGGCAATGGCTTCCCGAGCTTGCGAGATT
GCCAACTGAATGGATCCATCATCCATGGGACGCTCCTTTAACCGTACTCAAAGCTTCTGGTGT
GGAACTCGGAACAAACTATGCGAAACCCATTGTAGACATCGACACAGCTCGTGAGCTACTAG
CTAAAGCTATTTCAAGAACCCGTGAAGCACAGATCATGATCGGAGCAGCAGGCAGCGGCAG
CGGCAGCGTCGACACCGTGACCATCTTTGTGGCGGGAGTGCTCACCGCCGAGCTCACCATC
TGGAAGAAGATGGGCTGA

Supplementary Table 2. Experimental ingredients

Antibodies			
**Product Name**	**Supplier**	**Identifier**	**Concentration**
Anti-Bax monoclonal antibody	Santa Cruz Biotechnology	sc-7480	1:200
Anti-Tomm20 polyclonal antibody	Abcam	ab78547	1:1000
Recombinant Anti-Bax antibody	Abcam	ab182733	1:1000
mCherry (E5D8F) Rabbit mAb	Cell Signaling Technology	43590S	1:500
DsRed Antibody (E-8)	Santa Cruz Biotechnology	sc-390909	1:200
GFP Antibody (B-2)	Santa Cruz Biotechnology	sc-9996	1:200
Apaf-1 (D5C3) Rabbit mAb	Cell Signaling Technology	8969	1:200
Cleaved Caspase-3 (Asp175) Antibody	Cell Signaling Technology	9661	1:200
Cytochrome c (6H2.B4) Mouse mAb	Cell Signaling Technology	12963	1:200
Goat anti-Mouse IgG (H+L) Highly Cross-Adsorbed Secondary Antibody Alexa Fluor 488	Invitrogen	A11001	1:200
Goat anti-Rabbit IgG (H+L) Highly Cross-Adsorbed Secondary Antibody Alexa Fluor 568	Invitrogen	A11011	1:200
Goat anti-Rabbit IgG (H+L) Cross-Adsorbed Secondary Antibody Alexa Fluor™ 350	Invitrogen	A-11046	1:200
Goat anti-Mouse IgG (H+L) Cross-Adsorbed Secondary Antibody Alexa Fluor™ 350	Invitrogen	A-11045	1:200
Mounting medium with DAPI	Ibidi	50011	Dropwise
**Chemicals**			
Dimethyl Sulfoxide (DMSO)	Sigma Aldrich	317275	
Cisplatin	Sellekchem	SELLECK_S1166	
**Plasmids**			
Tomm20-Cib1-GFP	Addgene	#117242	
DBT^{S184E}	This study		
**Software**			
GraphPad Prism	GraphPad Software		
ImageJ	National Institutes of Health		
AlphaFold2	neurosnap.ai/		
RCSB PDB	rcsb.org		

Supplementary Figure 2. Blue light chamber design (a–g) A custom-built light irradiation box with a 450 nm LED board was generated. Polystyrene covers placed on top of the LEDs acted as illumination surfaces for the culture plates. (h) Transfected cells were prepared according to the written procedures. Twenty-four hours after seeding on a plate, the cells were transfected with plasmids. FAD was added to cells 24 h post-transfection. Finally, 48 h post-transfection, the cells were irradiated with blue light in the light chamber.

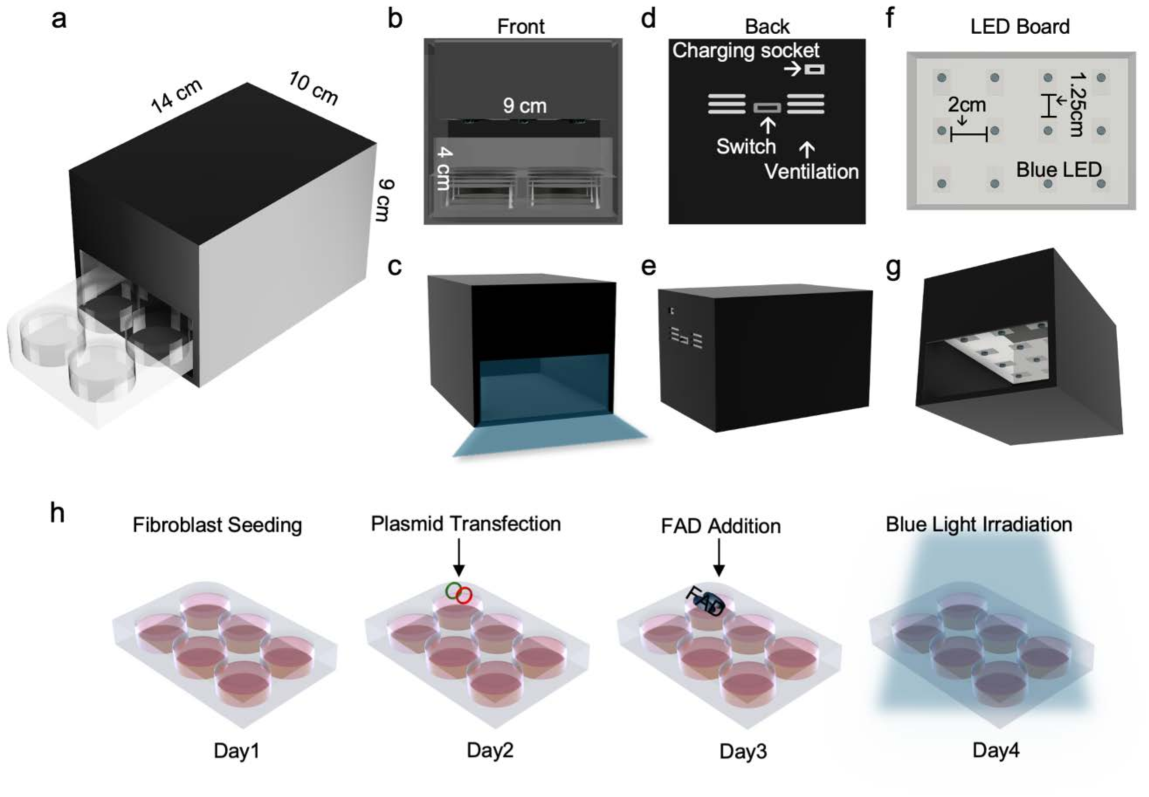

Supplementary Figure 3. Stoichiometry of rBAX (a) The rBAX:rTOMM20 transfection ratio was varied from 0:10 to 7:3, and its effects on endo-BAX and endogenous TOMM20 (endo-TOMM20) were examined by immunolabeling fixed cells after blue light irradiation. (b) Statistical analysis showed a significant increase in colocalization between the two proteins when the rBAX:rTOMM20 ratio ranged from 3:7 to 6:4 ($n = 5$ per group; one-way ANOVA with Dunnett's multiple comparisons test; ns, not significant ($p = 0.0911$); ****$p < 0.0001$; **$p = 0.0017$. The fluorescence intensity of BAX increased in proportion to the rBAX ratio, and this increase became significant only when the ratio was higher than 50 % compared to non-transfection of rBAX ($n = 9$ for all, one-way ANOVA with Dunnett's multiple comparisons test; ns, not significant ($p = 0.4730$ and $p = 0.7404$, respectively); *$p = 0.0240$; **$p = 0.0013$; ***$p = 0.001$).

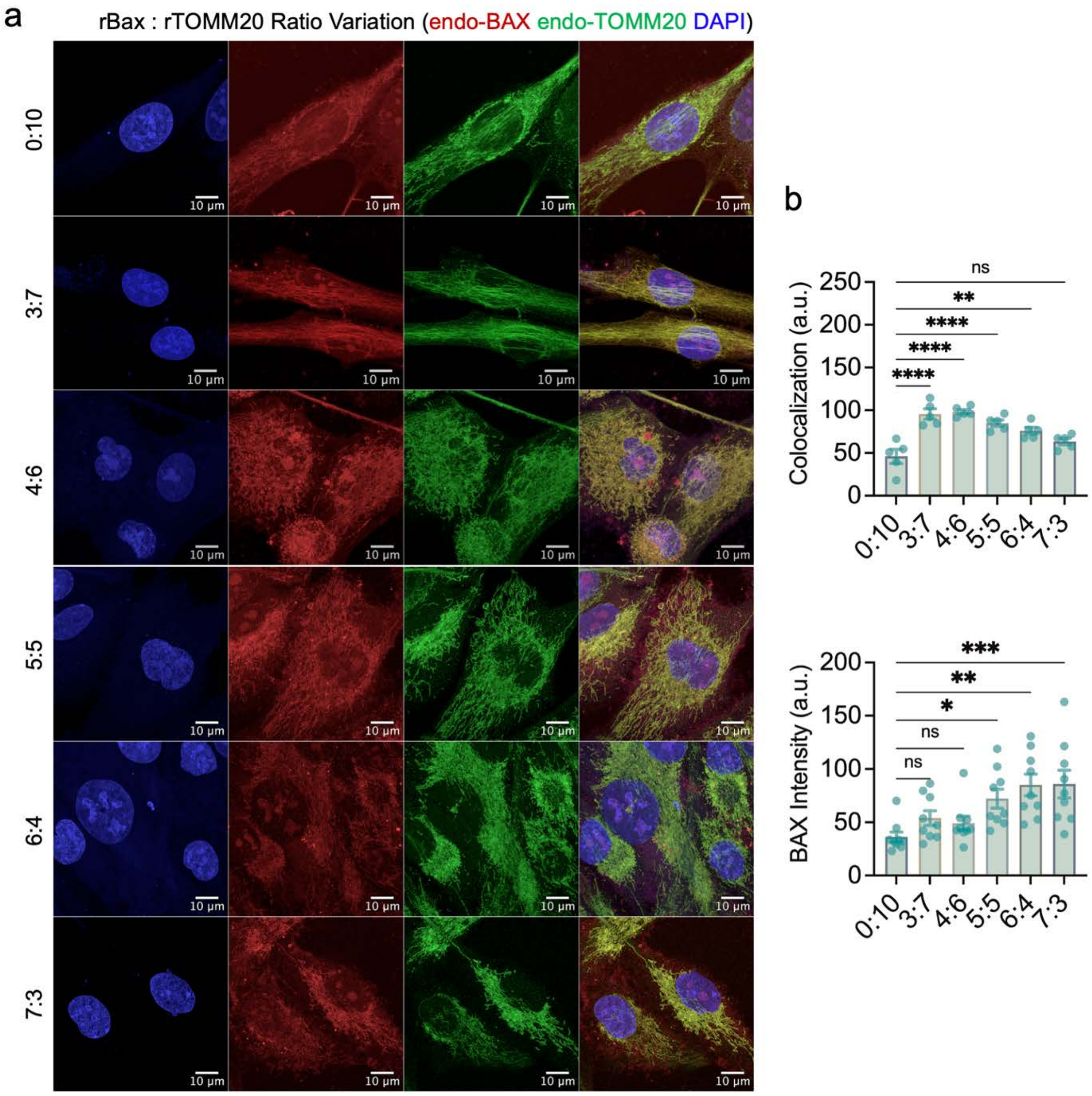
a
rBax : rTOMM20 Ratio Variation (endo-BAX endo-TOMM20 DAPI)
0:10
3:7
4:6
5:5
6:4
7:3
10 μm
b
Colocalization (a.u.)
BAX Intensity (a.u.)
ns
**
****
*
***
0:10
3:7
4:6
5:5
6:4
7:3
250
200
150
100
50
0

Supplementary Figure 4. Effect of a single light pulse (a) DBT was tracked under a confocal microscope to confirm whether 0.2 mW of 488 nm laser power (measured at the laser port) induces noticeable rBAX aggregation within 5 minutes. The light pulse was delivered to the cell 10 sec after acquiring 5 consecutive frames. (b) Mitochondrial morphology at the irradiation site was examined before and after 5 minutes of light exposure. No evident changes were observed in either mitochondrial morphology or dynamics. (c) rBAX distribution was analyzed by frame profile, showing no significant increase in fluorescence intensity (grey area: dark incubation; blue line: 488 nm light irradiation).

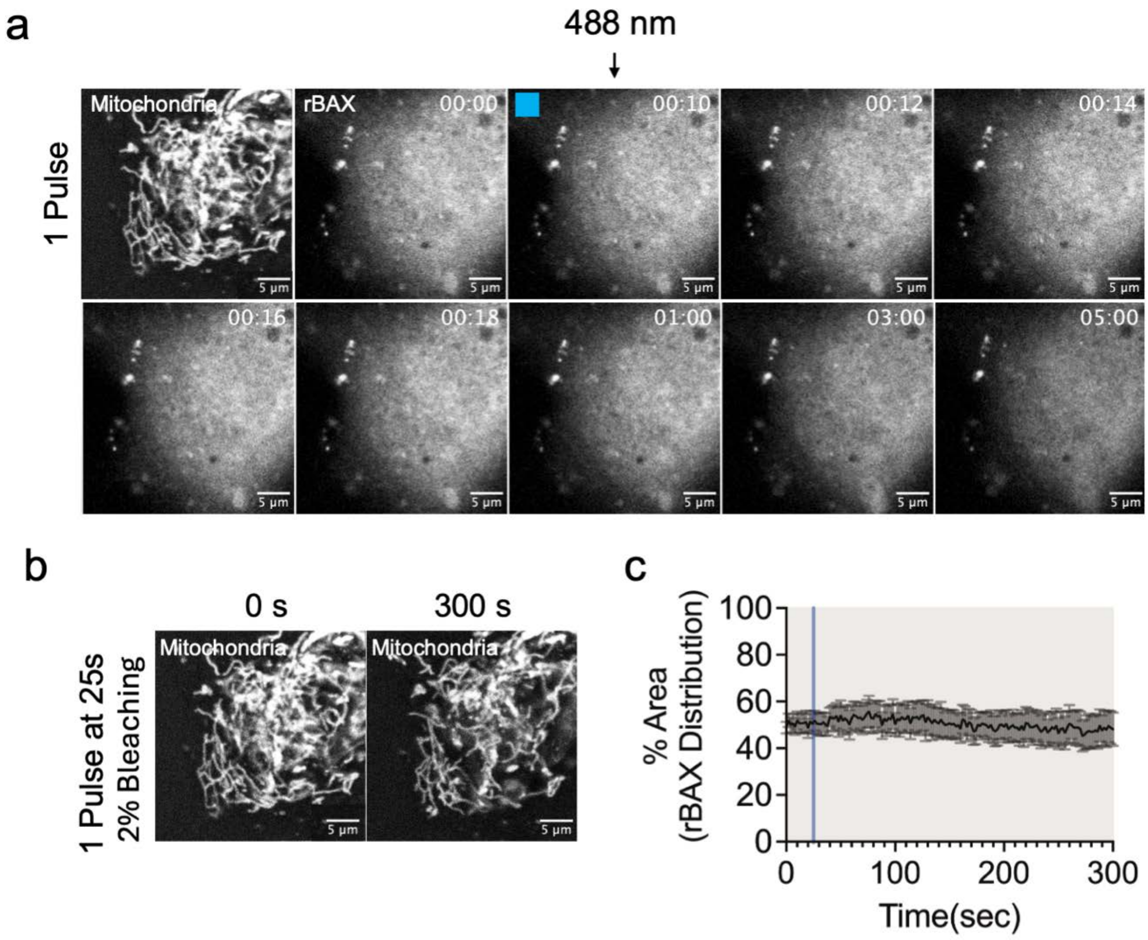

Supplementary Figure 5. Temporal kinetics of rBAX association and dissociation under light stimulation (a) Live-cell imaging of HBT- and DBT-transfected fibroblasts under iterative light stimulation followed by dark incubation. While HBT and DBT both showed gradual aggregation upon repeated stimulation, DBT exhibited earlier redistribution dynamics. (b) Time-lapse images of HBT and DBT association and dissociation events in subregions. DBT showed progressive dissociation during ongoing light stimulation, whereas HBT dissociation occurred only after the termination of light exposure (light termination at 11 min 25 sec). (c–d) Quantification of fluorescence profiles at each time point for the representative images. Association onset was defined as the first observation of fluorescent nucleation, and dissociation onset was defined as the time point of a 50 % reduction in protein aggregate size. Average association onset was similar for HBT (83.00 ± 22.64 sec, n = 5) and DBT (81.25 ± 80.71 sec, n = 5). However, dissociation kinetics differed markedly: HBT dissociated only after the light was turned off (831.00 ± 154.80 sec after association onset, n = 5), while DBT dissociation initiated during light exposure (255.00 ± 203.85 sec after association onset, n = 5).

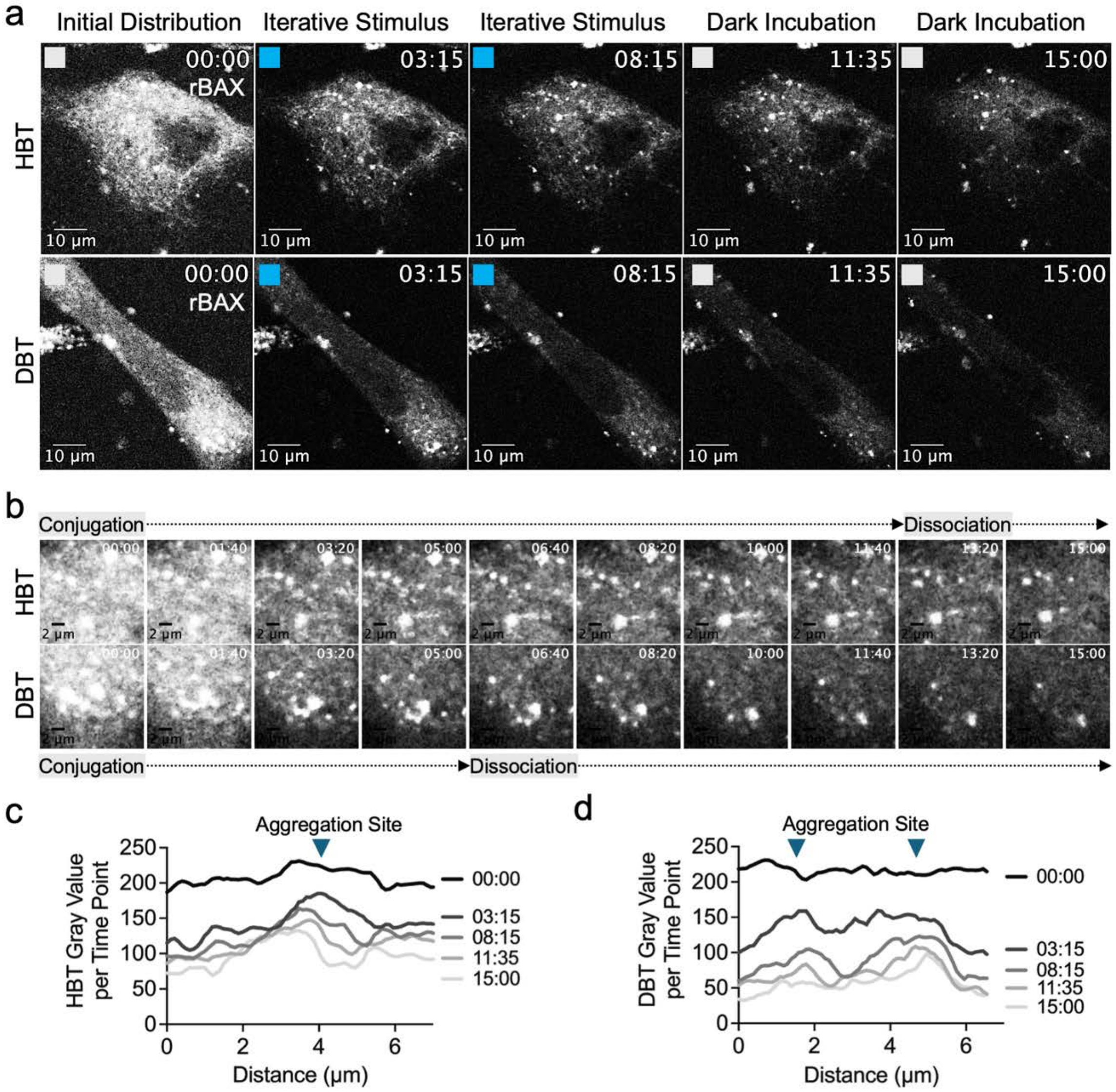

Association onset	83 ± 22.64 (sec)
Dissociation onset - Association onset	831.00 ± 154.80 (sec)

Association onset	81.25 ± 80.71 (sec)
Dissociation onset - Association onset	255.00 ± 203.85 (sec)

Supplementary Figure 6. Phototoxicity evaluation under confocal microscopy (a) Cells transfected with rTOMM20 alone were used as controls to assess light-induced toxicity. Cells were monitored for 900 sec from the onset of light exposure, while varying laser intensity, exposure interval, and frame time (laser output power measured at the laser port: 10 mW). (b) Photobleaching was calculated as the fractional loss of fluorescence signal using the formula $(F_0\text{-}F_t)/F_0$ (F_t=fluorescence at time t; F_0= initial fluorescence). In this graph, higher values indicate greater photobleaching. (c) Mitochondrial fission (indicative of cell death) and photobleaching were assessed for each condition. At 0.2 % power (0.02 mW; interval, 5 s; pixel time, 3.81 μs), neither fission nor bleaching was observed. At 1 % power with identical settings, photobleaching was evident. This effect was not alleviated by extending the light interval but was sensitive to pixel time. When pixel time was reduced to 1.40 μs, powers of 2–5 % (0.2–0.5 mW) did not induce fission or bleaching, whereas 10% power caused both. These results indicate that at laser powers up to 5 % (0.5 mW), pixel dwell time is a critical determinant of phototoxicity.

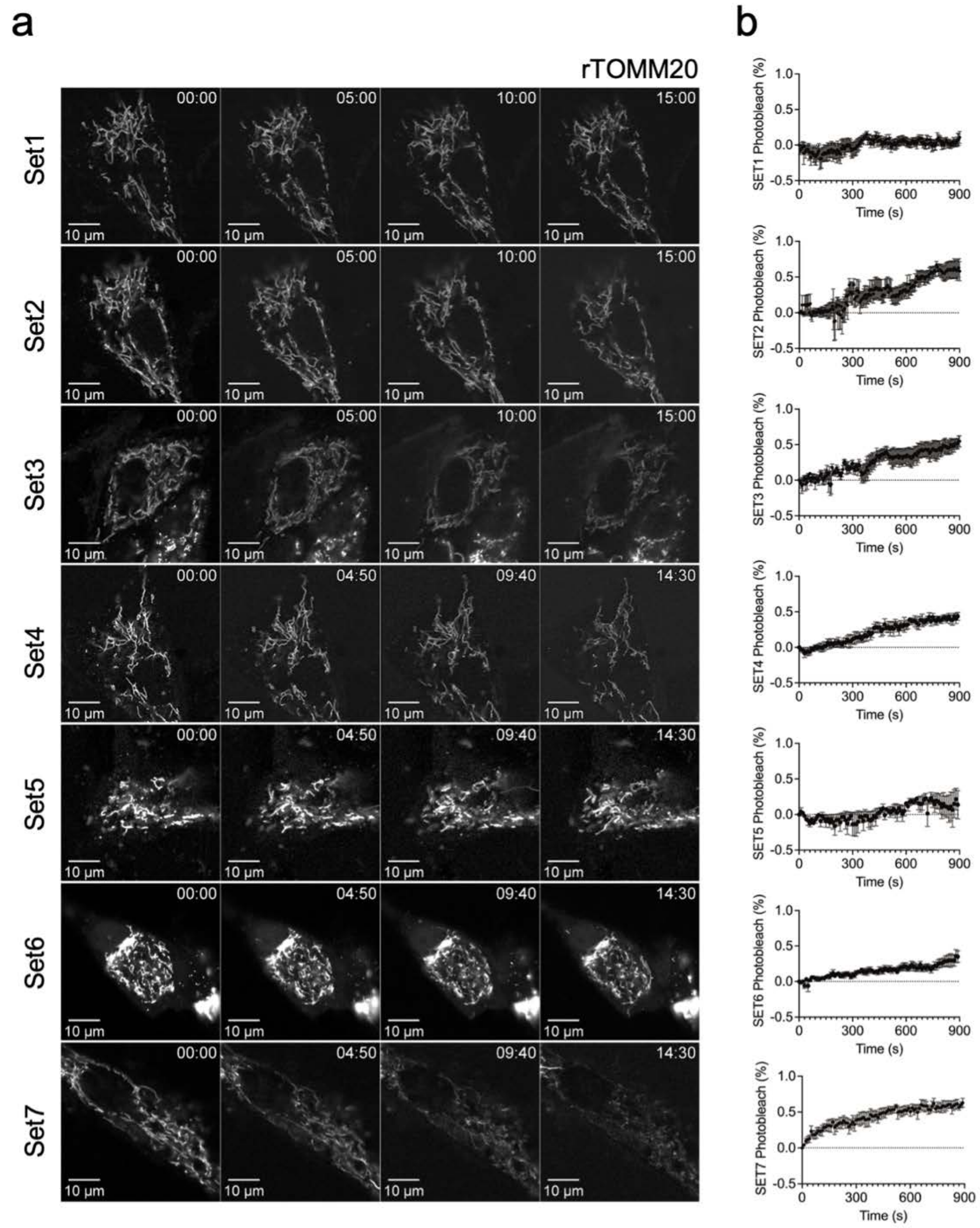

c

Set No.	Light Power	Recording Time	Light Interval	Frame Time	Pixel Time	Fission	Bleaching
1	0.2 %	15 min	5 sec	3.81 μs	1.37 μs	X	X
2	1 %	15 min	5 sec	3.81 μs	1.37 μs	X	O
3	2 %	15 min	5 sec	3.81 μs	1.37 μs	X	O
4	2 %	15 min	10 sec	3.81 μs	1.37 μs	X	O
5	2 %	15 min	10 sec	1.40 μs	1.01 μs	X	X
6	5 %	15 min	10 sec	1.40 μs	1.01 μs	X	X
7	10 %	15 min	10 sec	1.40 μs	1.01 μs	O	O

Supplementary Figure 7. Introduction of point mutation in rBAX (a) Wild-type rBAX induces autoactivation and apoptosis in the absence of light. (b) The S184E point mutation inhibits rBAX autoactivation, thereby preventing apoptosis under dark conditions.

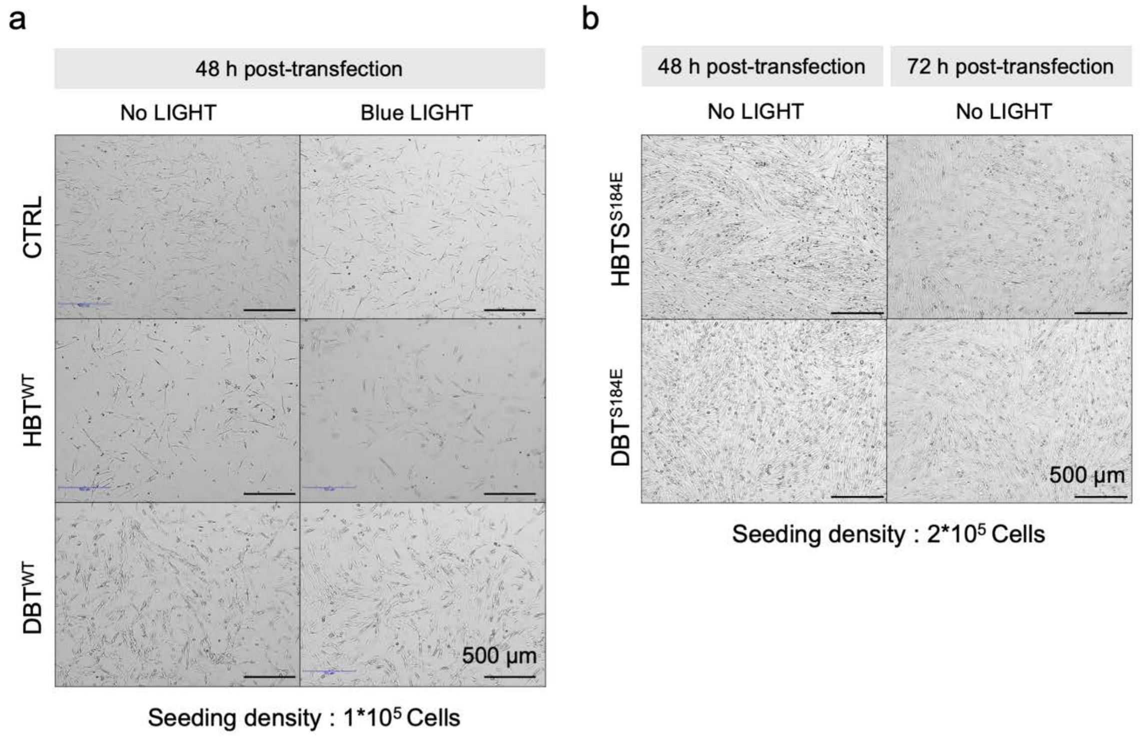

Supplementary Figure 8. 3D-rendered images of rBAX-rTOMM20 conjugated structures from multiple perspectives endo-BAX is shown in red, rBAX in yellow, and rTOMM20 in cyan. These images illustrate the spatial distribution and interactions among these mitochondrial proteins under each condition: (a) In the CTRL condition (rTOMM20 only), endo-BAX displayed a membrane-embedded conformation. From bottom-view images highlighting endo-BAX's penetration into the MOM, multiple endo-BAX molecules were observed to be deeply embedded. (b-c) HBT- and DBT-transfected fibroblasts. Under the HBT condition, large complexes formed among endo-BAX, rBAX, and rTOMM20. Top-view images revealed extensive endo-BAX conjugation with the HBT complex, generating a sizeable aggregate (1.483 μm) on the mitochondria. In bottom and side views, endo-BAX showed deep penetration alongside the rBAX cluster, while DBT showed active interaction between rBAX and rTOMM20 without massive recruitment of endo-BAX. Scale bar: 1 μm

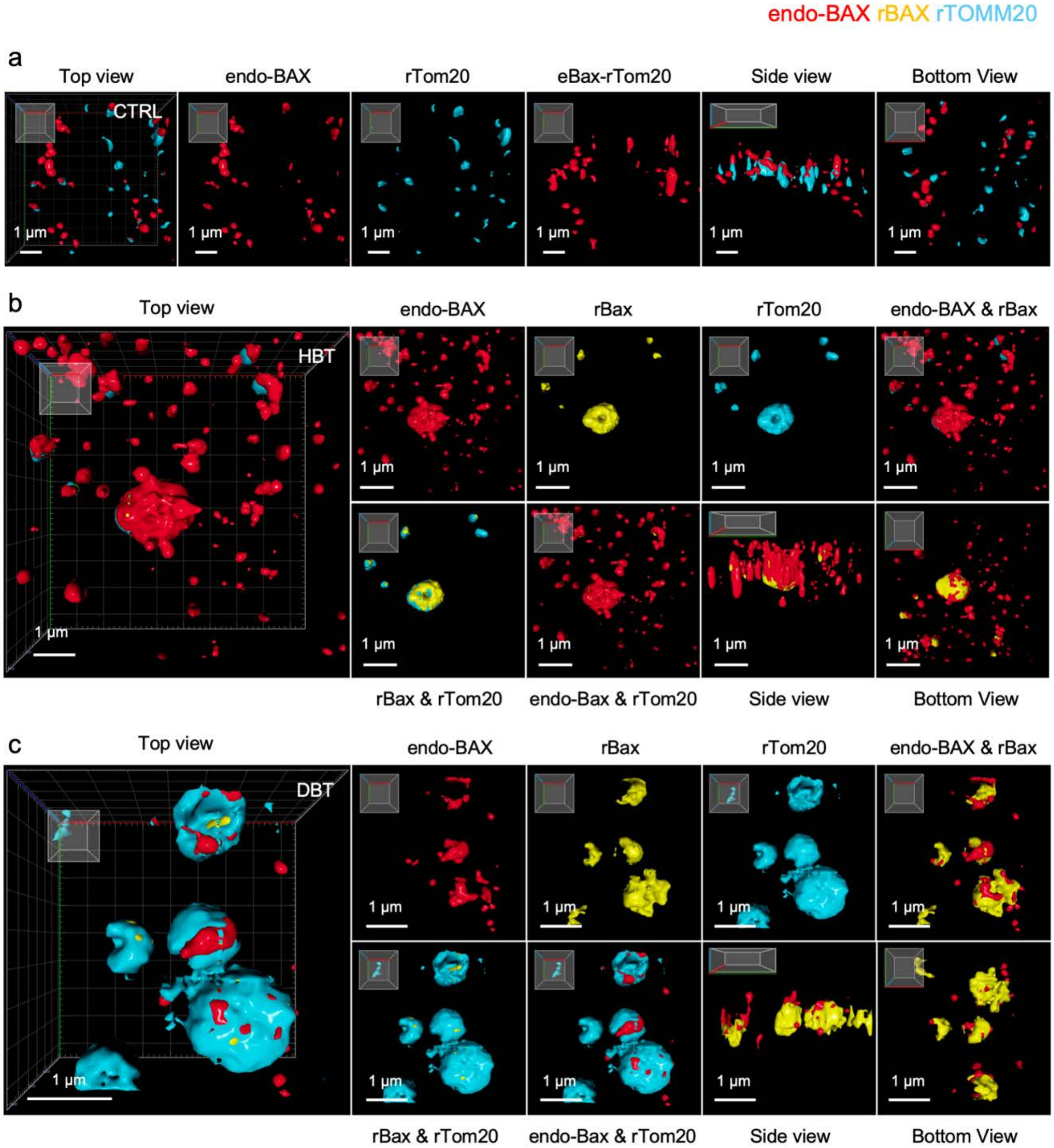
endo-BAX rBAX rTOMM20
a
Top view
endo-BAX
rTom20
eBax-rTom20
Side view
Bottom View
CTRL
1 µm
b
Top view
endo-BAX
rBax
rTom20
endo-BAX & rBax
HBT
rBax & rTom20
endo-Bax & rTom20
Side view
Bottom View
c
Top view
endo-BAX
rBax
rTom20
endo-BAX & rBax
DBT
rBax & rTom20
endo-Bax & rTom20
Side view
Bottom View

Supplementary Figure 9. Quantification of average gray values for the indicated cellular components (a–d) Average intensity profiles derived from multiple ROIs under each condition. The alignment of intensity peaks demonstrates the co-localization of the three molecules at the same x-axis distance. Red, cyan, and yellow lines represent the intensities of endo-BAX, rTOMM20, and rBAX, respectively (Mean ± SEM).

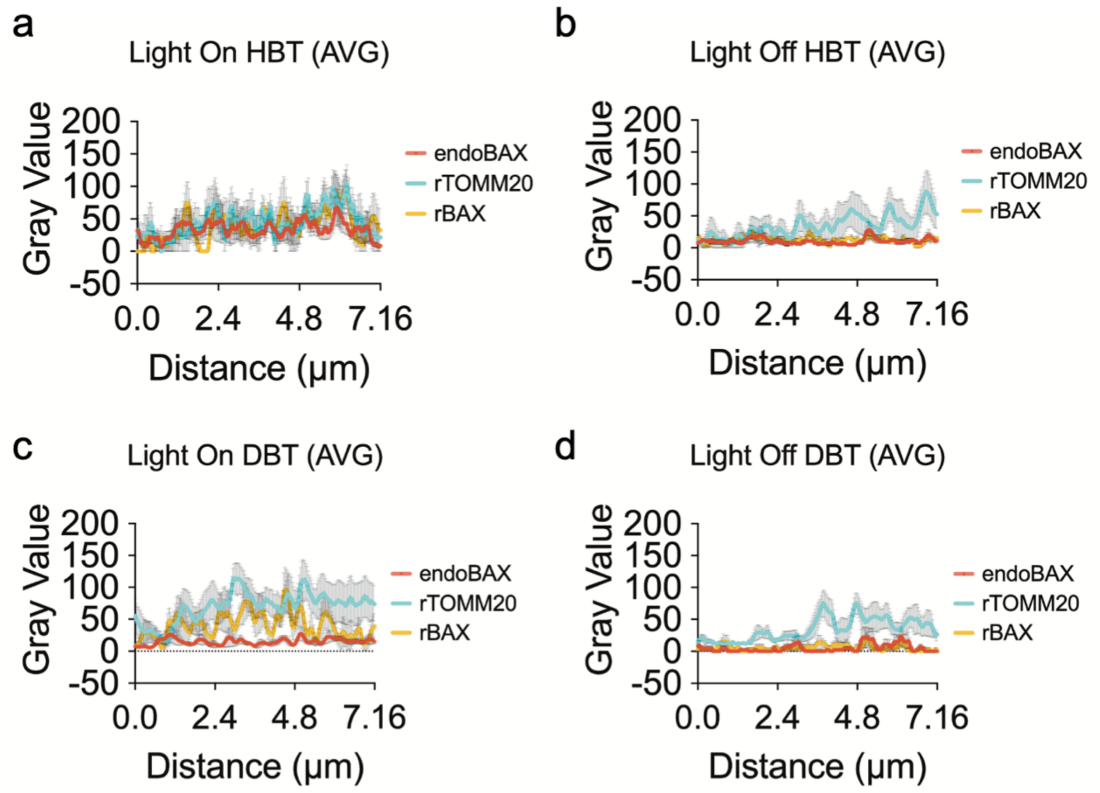

Supplementary Figure 10. Co-localization analysis Immunostained images were color-thresholded to highlight specific regions: red and green indicate individual targets, while orange (overlay regions) represents areas of co-localization. The thresholded areas are shown in white. Consistent settings were applied across all experimental groups.

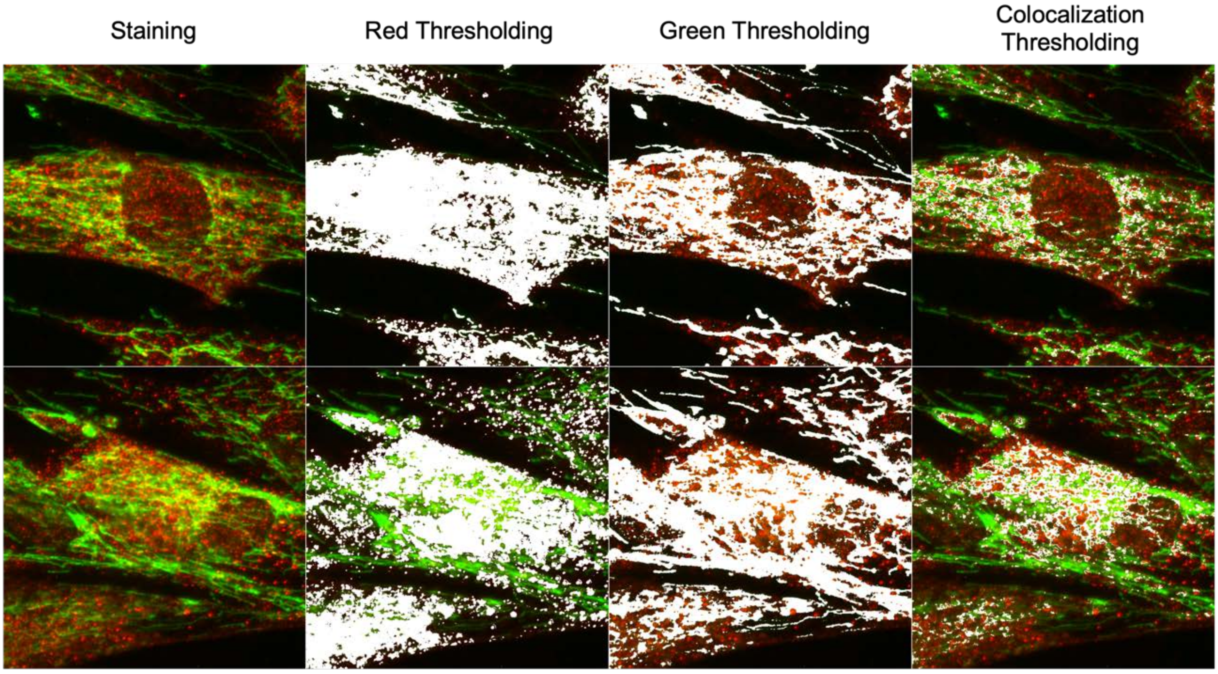

Supplementary Figure 11. BAX size analysis To measure BAX aggregation size, immunostained 8-bit images were thresholded using the Shanbhag method in ImageJ. Particles were filtered based on size and counted, while highly saturated areas were excluded from the analysis. Consistent settings were applied across all experimental groups.

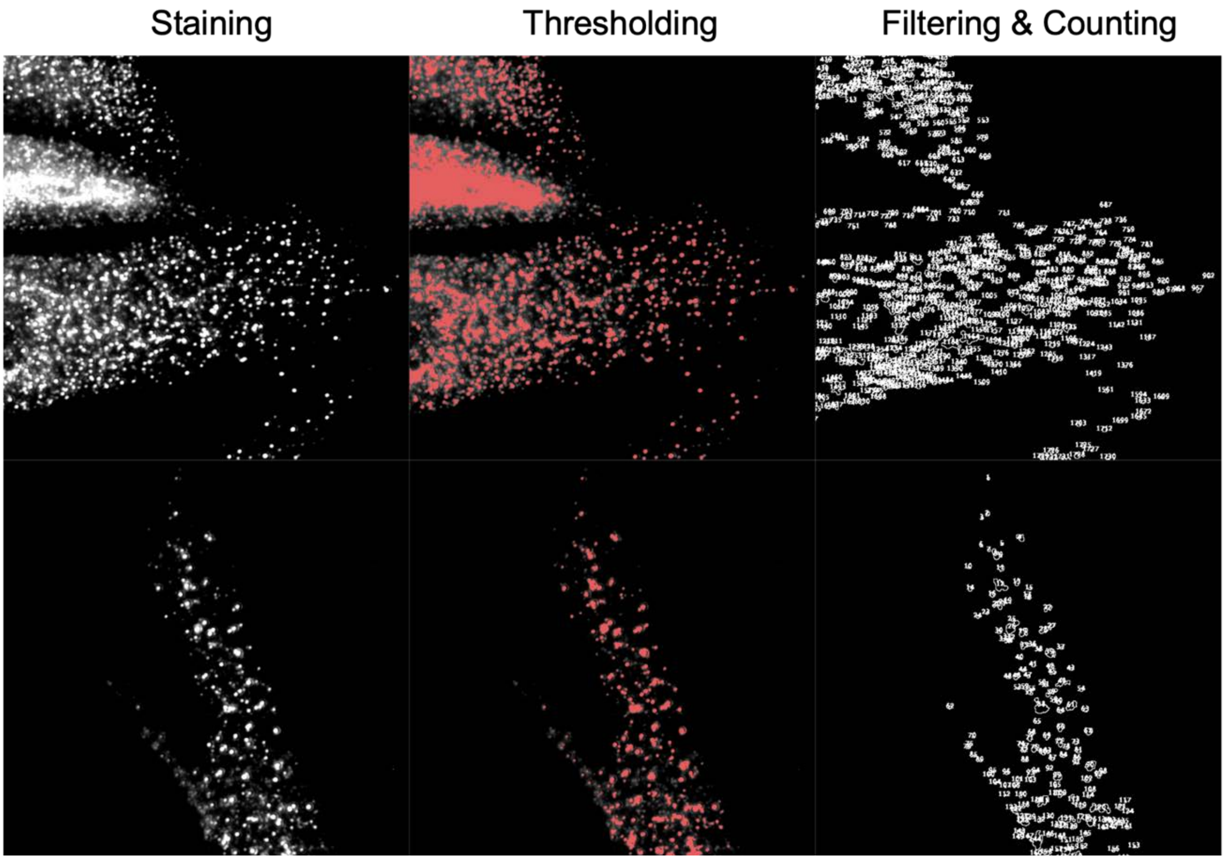

Supplementary Figure 12. Mitochondrial fission analysis To analyze mitochondrial fission, immunostained 8-bit images were thresholded using the MaxEntropy method in ImageJ. Particles were filtered based on size and counted. Consistent settings were applied across all experimental groups.

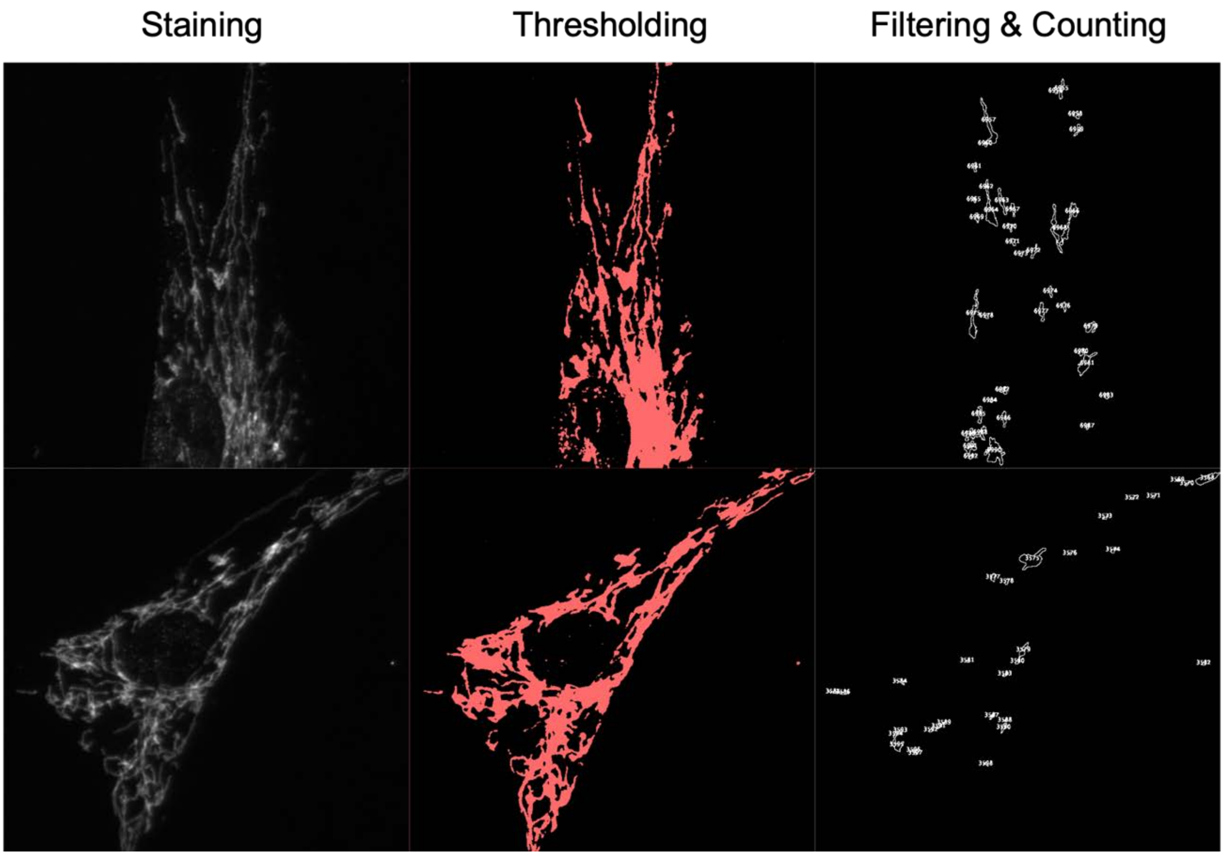

Supplementary Figure 13. Merged immunostained images (dark condition) Cells maintained in the dark (without blue light irradiation) were fixed 48 h post-transfection and stained with anti-BAX and anti-TOMM20 antibodies to assess cellular morphology and subcellular localization. Scale bar, 100 μm. Red, endo-BAX; Green, endo-TOMM20; Blue, DAPI.

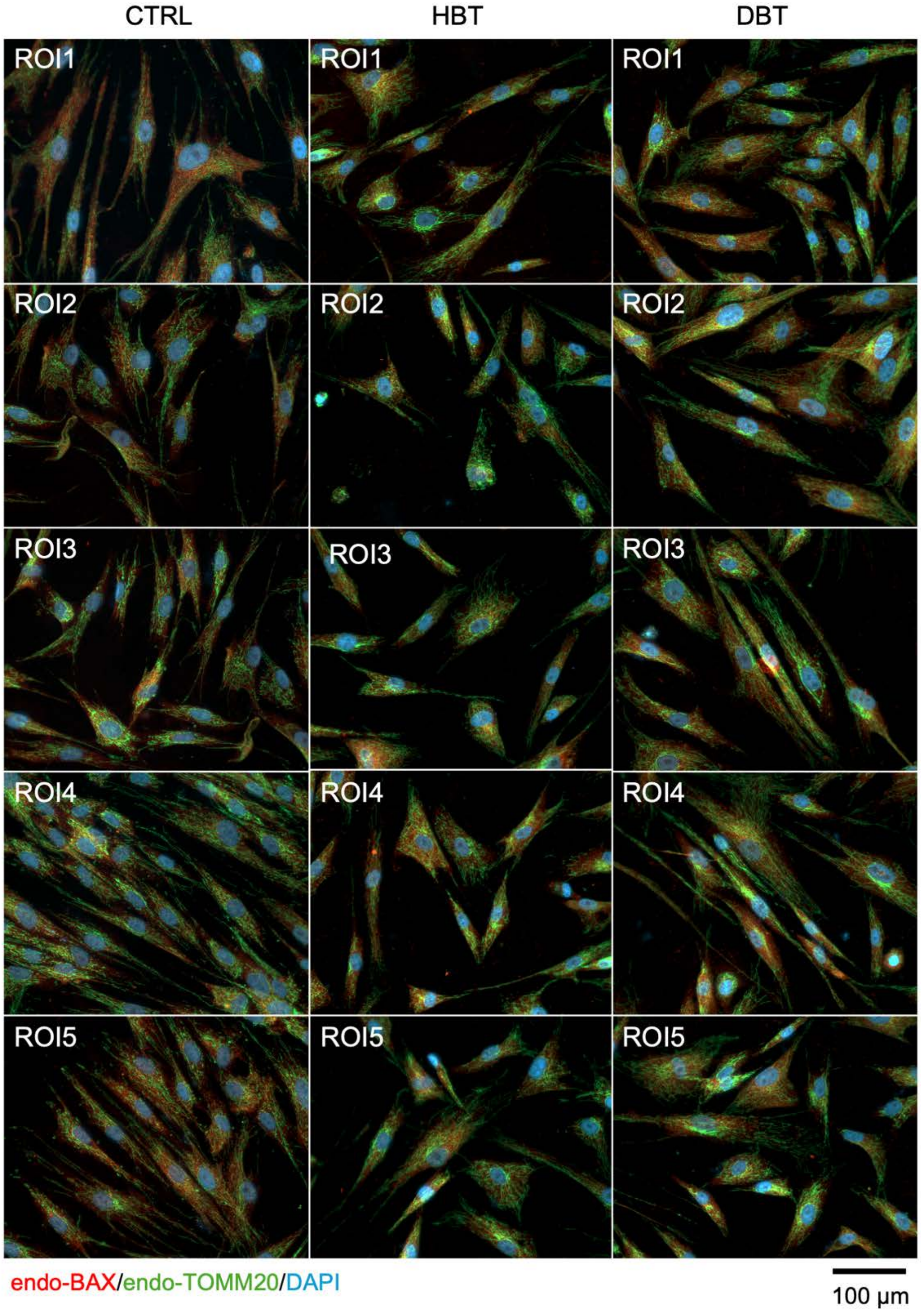

Supplementary Figure 14. Immunostained images and analysis of endo-BAX (dark condition) (a) Dark-incubated cells without blue light irradiation were stained with anti-BAX antibody to measure aggregation size. Scale bar: 20 μm; Red: BAX; Blue: DAPI. (b, c) Bar graphs showing BAX aggregation size and perimeter as measured by fluorescent puncta in each cell (n = 300 per group). Data were analyzed using one-way ANOVA with Tukey's multiple comparisons test ($p = 0.2231$ and $p = 0.3259$, respectively). ns, non-significant; CTRL, rTOMM20-transfected fibroblasts; HBT, HBT-transfected fibroblasts; DBT, DBT-transfected fibroblasts. All data are shown as mean ± SEM.

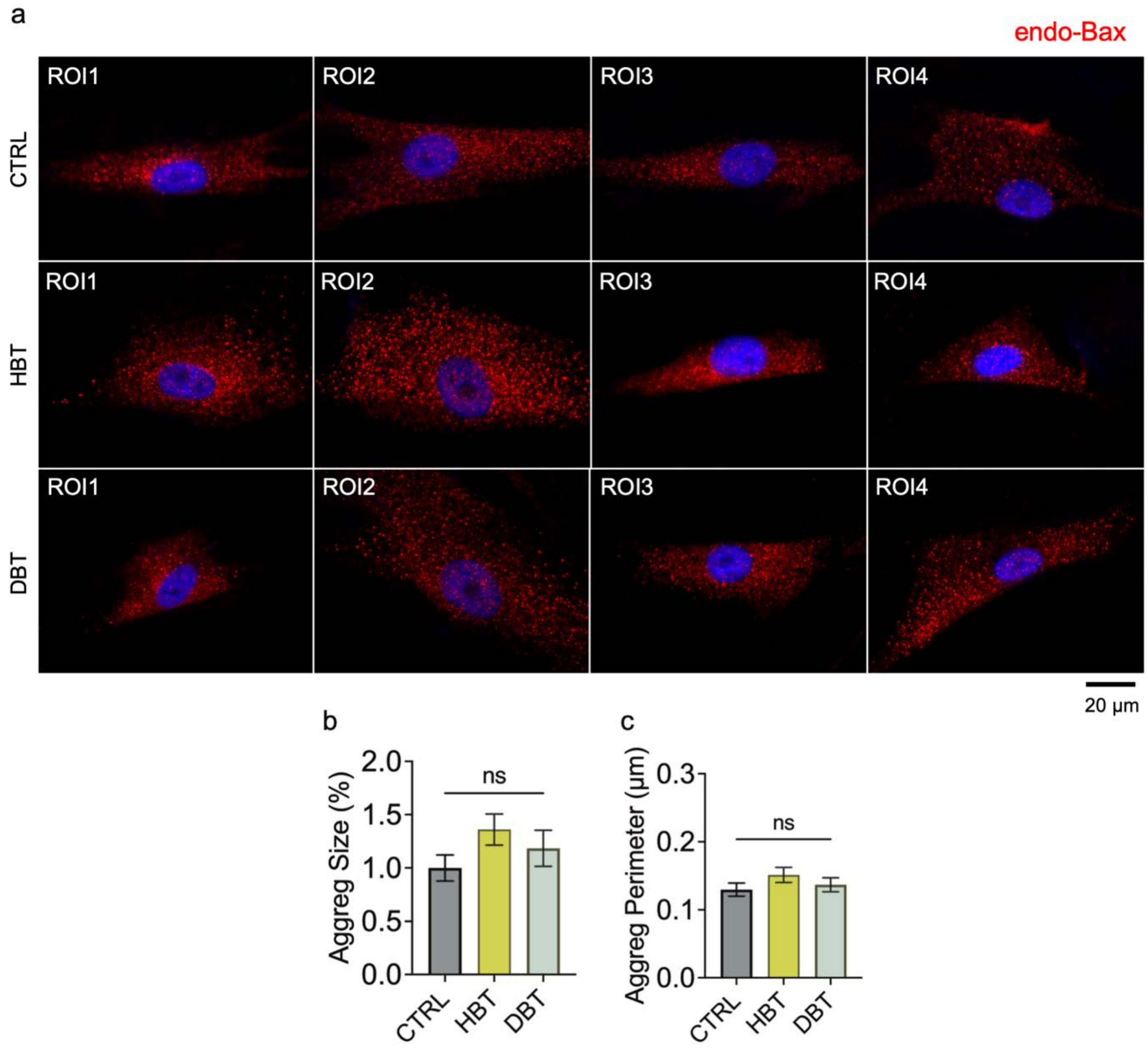

Supplementary Figure 15. DAPI staining and analysis (dark condition) (a) Cells maintained in the dark (without blue light irradiation) were stained with DAPI to assess cell number and nuclear morphology. Scale bar: 100 μm. (b) Quantification of cell number based on DAPI counts per ROI (n = 8 per group; p = 0.1031). (c) Analysis of nuclear solidity (n = 40 per group; p = 0.7897). Data were analyzed using One-way ANOVA followed by Tukey's multiple comparisons test. ns, non-significant. CTRL, rTOMM20-only transfected fibroblasts; HBT, HBT-transfected fibroblasts; DBT, DBT-transfected fibroblasts. Data represent mean ± SEM.

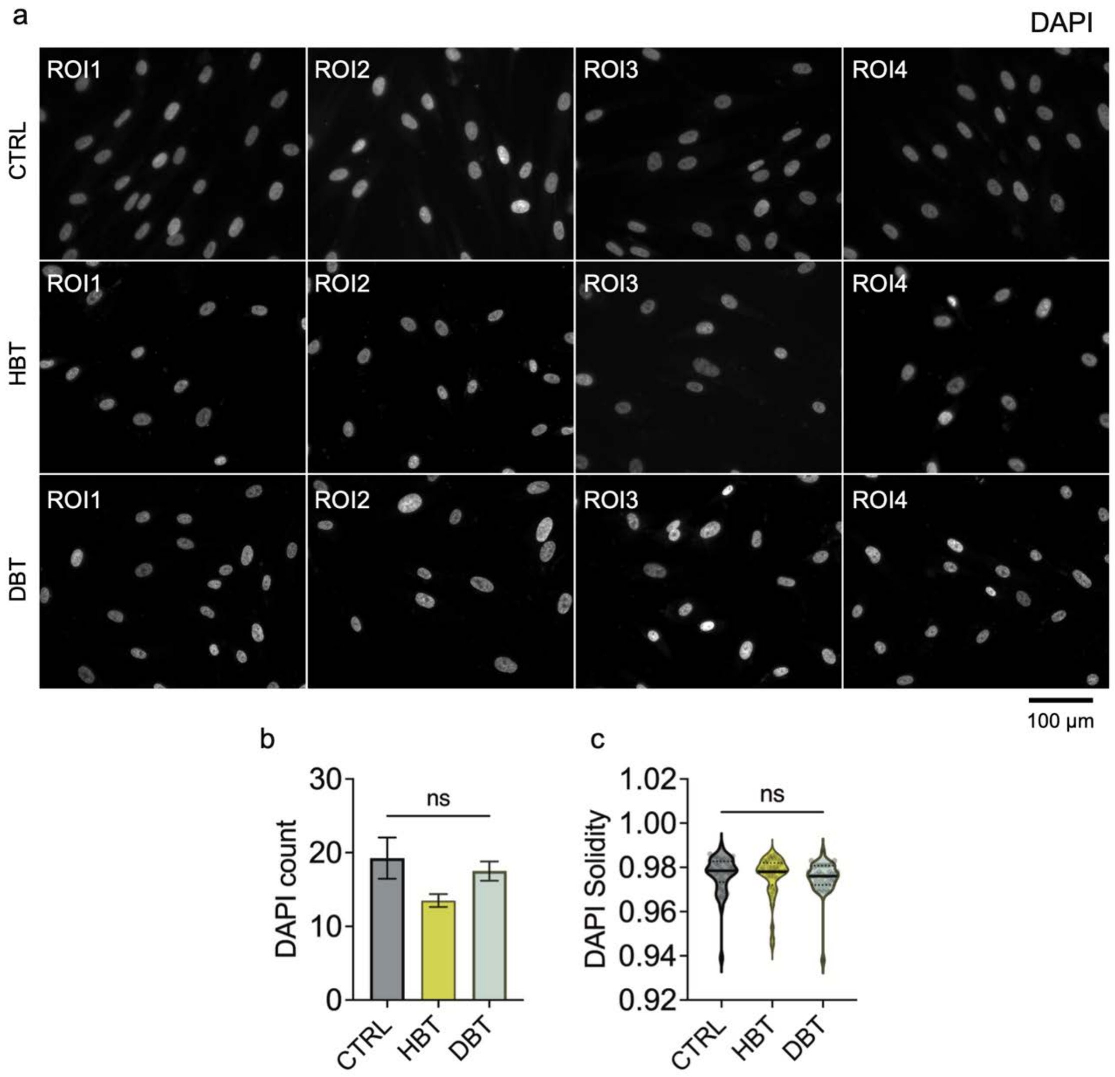

Supplementary Figure 16. Immunostained images of endo-TOMM20 and analysis (dark condition) (a) Dark-incubated cells without blue light irradiation were stained with anti-TOMM20 antibody to visualize endo-TOMM20 for mitochondrial fission analysis. Scale bar: 20 μm; Green: TOMM20; Blue: DAPI. (b–d) Bar graphs showing measurements of mitochondrial perimeter, aspect ratio, and circularity after dark incubation (n = 300 per group; one-way ANOVA with Tukey's multiple comparisons test; p = 0.2801, 0.3983, and 0.5501, respectively). ns, non-significant; CTRL, rTOMM20-transfected fibroblasts; HBT, HBT-transfected fibroblasts; DBT, DBT-transfected fibroblasts; mean ± SEM for all.

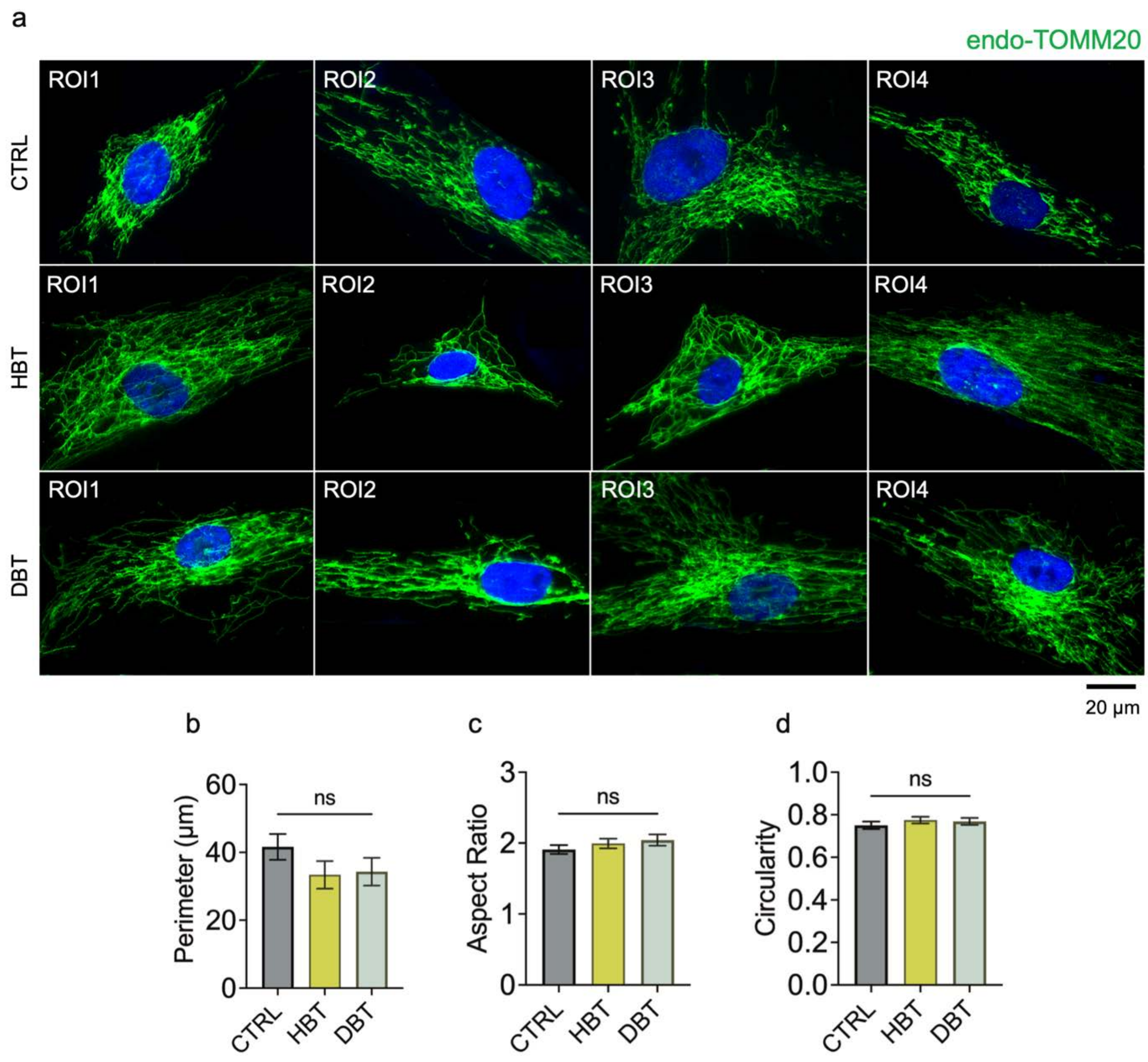

Supplementary Figure 17. Immunostaining and analysis of CytC distribution under blue light illumination (Cisplatin treated) (a) Cells exposed to blue light were stained for CytC and rBAX to assess their distribution and co-localization. Scale bar: 10 μm. (b, c) Intensity profiles of the boxed regions indicated by asterisks are shown. (d) Quantification of CytC integrity based on aspect ratio (AR) (n = 300 cells per group; p = 0.8716 for CTRL vs. HBT, p = 0.0391 for CTRL vs. DBT, and p = 0.0093 for HBT vs. DBT). Statistical significance was determined by one-way ANOVA followed by Tukey's post hoc test. *p < 0.05; **p < 0.01; ns, non-significant; mean ± SEM for all.

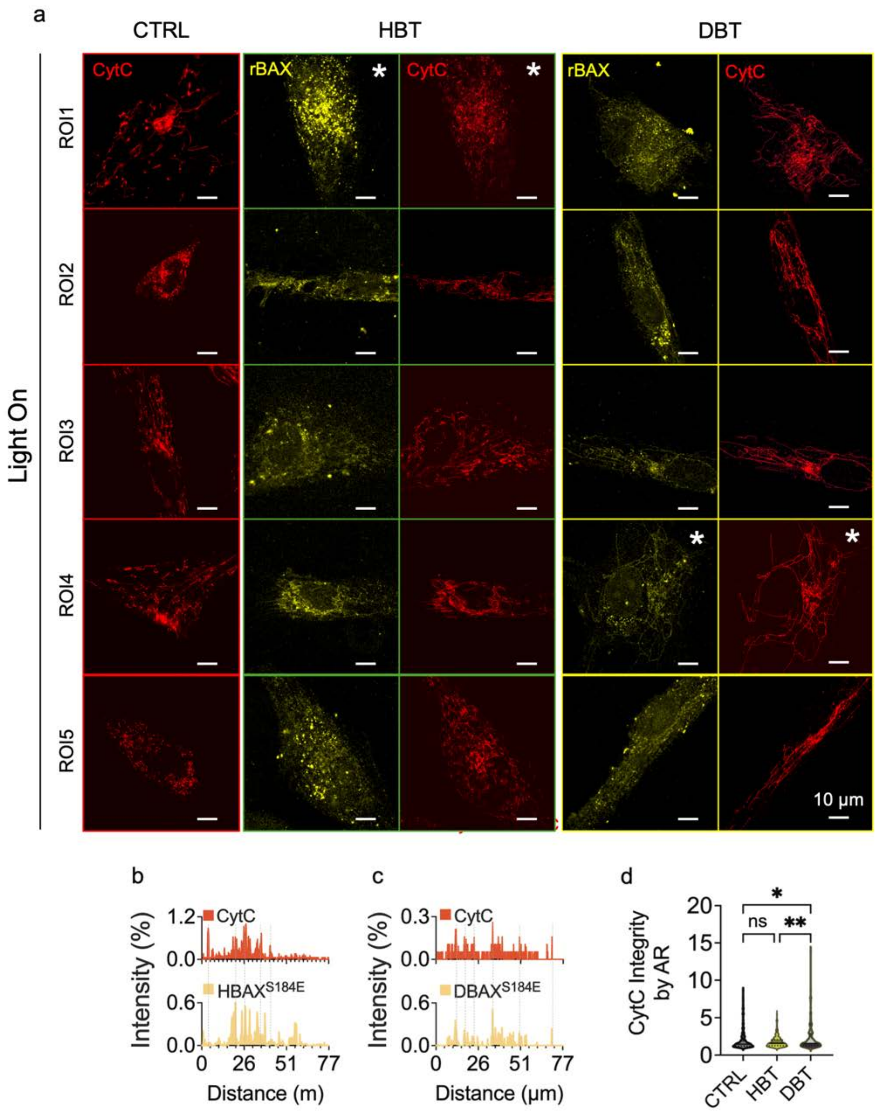

Supplementary Figure 18. Immunostaining and analysis of CytC in dark-incubated cells (Cisplatin treated) (a) Dark-incubated cells were stained with CytC and rBAX to evaluate their distribution and colocalization. Scale bar: 10 μm. (b) Bar graph illustrating CytC integrated density (n = 7 per group; one-way ANOVA with Tukey's post hoc test; p = 0.1514 for CTRL vs. HBT, p > 0.9999 for CTRL vs. DBT, and p = 0.1482 for HBT vs. DBT). ns, non-significant. (c) Bar graph presenting the morphological analysis of CytC integrity measured by aspect ratio (AR) (n = 300 per group; one-way ANOVA with Bonferroni's multiple comparisons test; p = 0.8068 for CTRL vs. HBT, p = 0.3934 for CTRL vs. DBT, and p = 0.7752 for HBT vs. DBT). ns, non-significant; mean ± SEM for all.

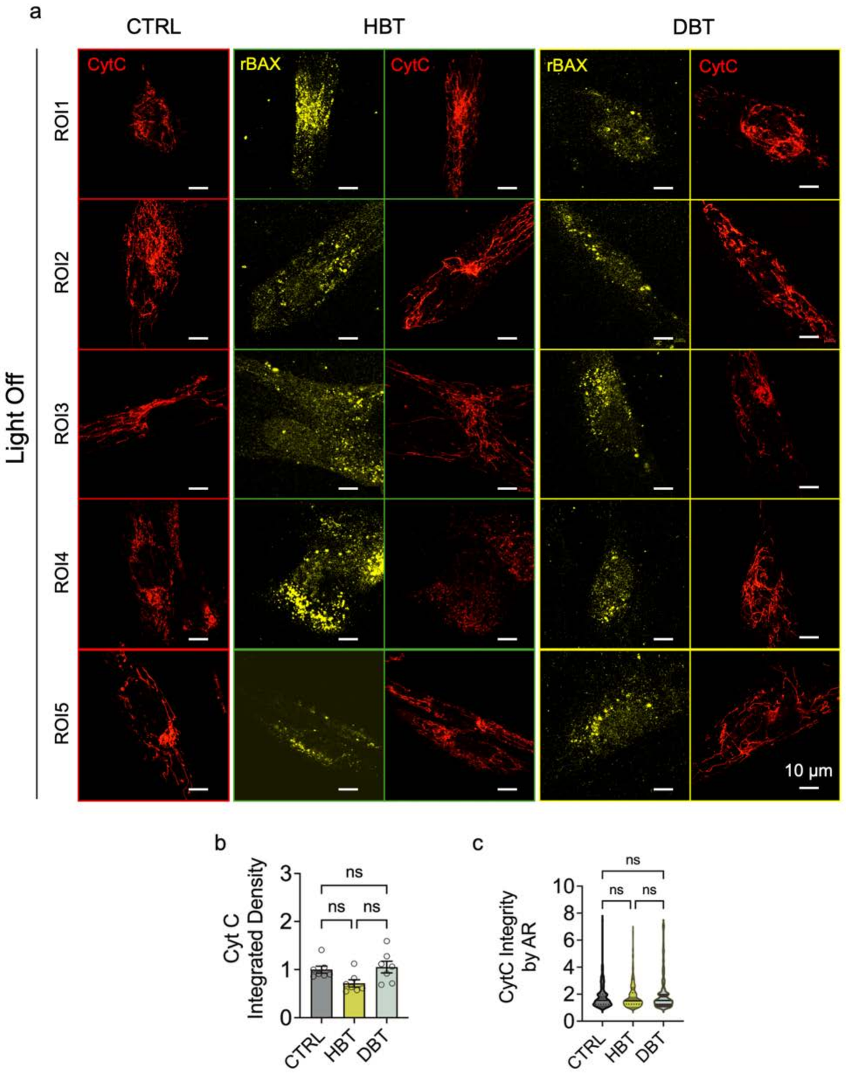

Supplementary Figure 19. 3D-rendered immunofluorescence images demonstrating the colocalization of CC3, rBAX, and rTOMM20 under Cisplatin treatment Merged images highlight the spatial association between apoptotic markers and mitochondrial structures under blue light irradiation. In these images, CC3 appears red, rBAX yellow, and rTOMM20 cyan. The panels display protein distribution in: (a) CTRL (rTOMM20-only), (b) HBT-transfected, and (c) DBT-transfected conditions. The DBT condition exhibited minimal CC3 signals and reduced co-localization with rBAX compared to the other groups.

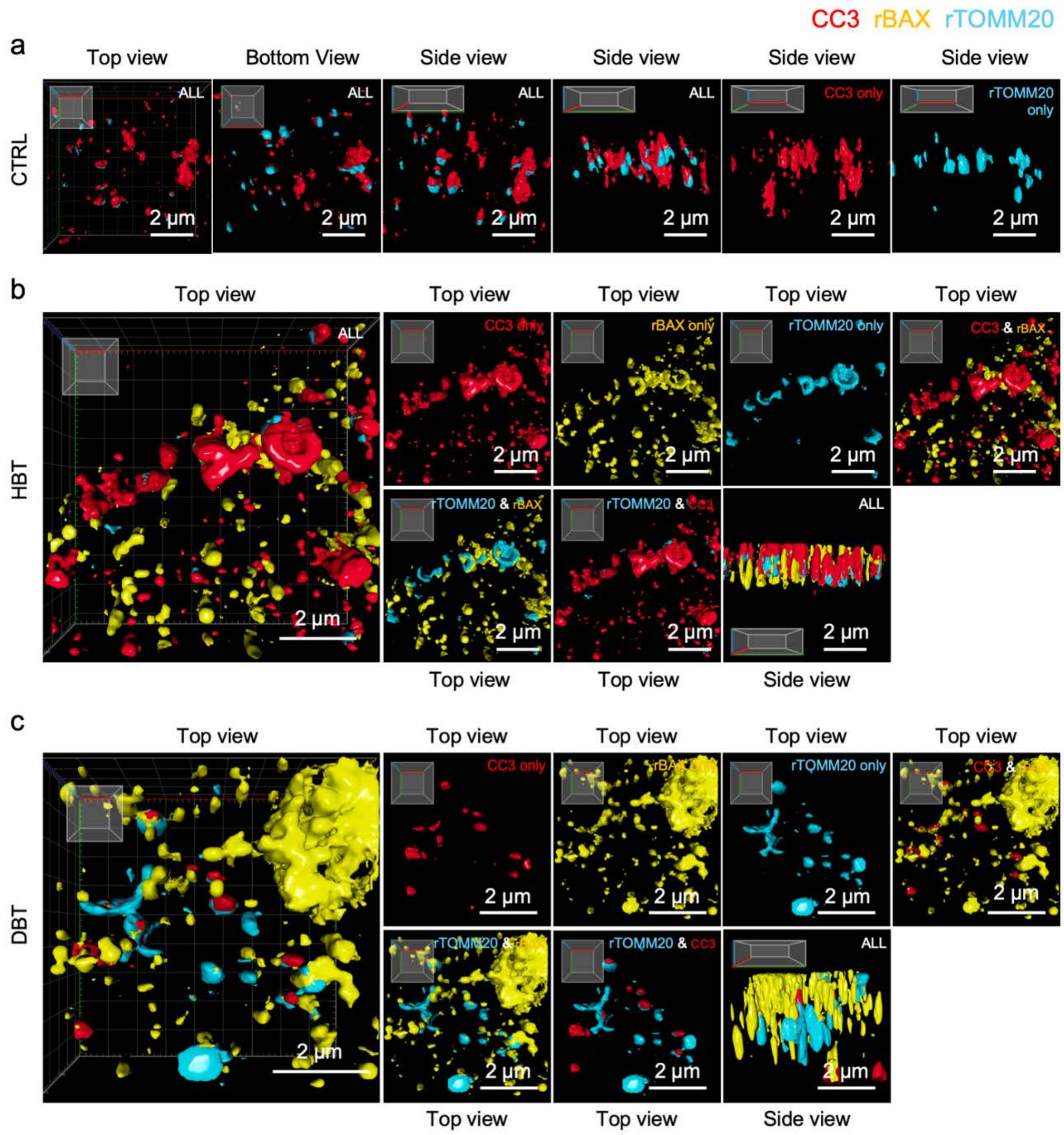

Supplementary Figure 20. Immunostained images of CC3 and analysis under blue light-incubated conditions (Cisplatin treated) (a) Blue Light-incubated cells were stained with CC3 and rBAX to evaluate their distribution and colocalization. Scale bar: 10 μm.

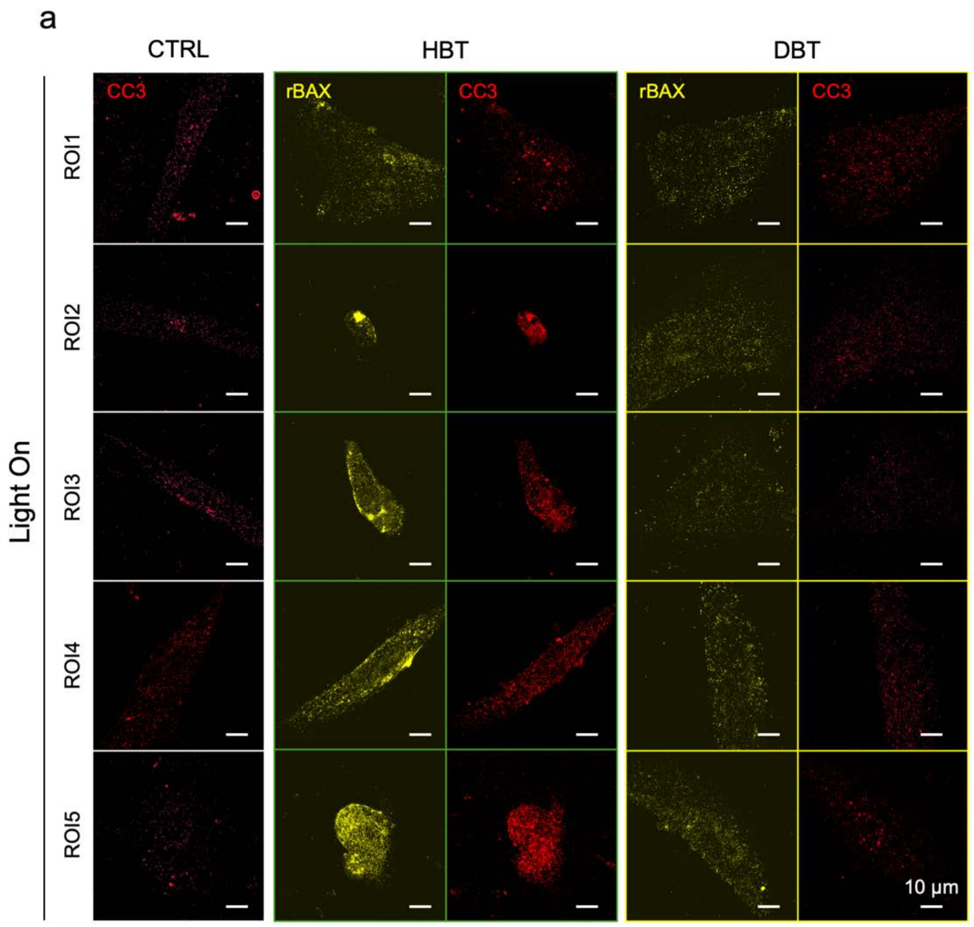

Supplementary Figure 21. Immunostained images of CC3 and analysis in dark conditions (Cisplatin treated) (a) Dark-incubated cells were stained with CC3 and rBAX to evaluate their distribution. Scale bar: 10 μm. (b) Correlation analysis of rBAX–CC3, showing reported R^2, slope, and p-values (n = 8 per group). The results indicate no significant distributional correlation between the two proteins. (c) Analysis of normalized CC3 levels per cell using one-way ANOVA with Tukey's post hoc test (n = 9 per group; p = 0.9932). ns, non-significant; mean ± SEM for all

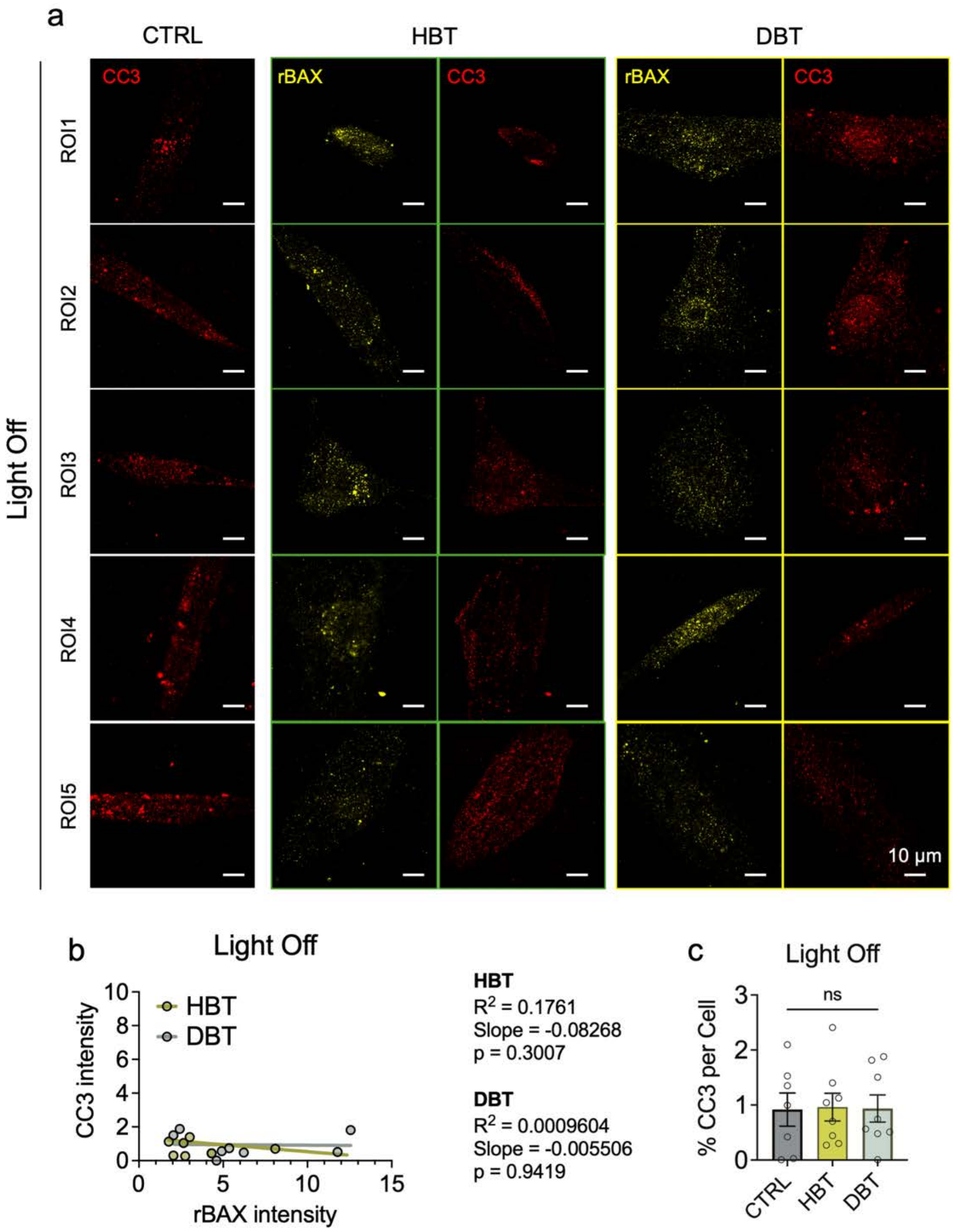

Supplementary Figure 22. Anti-apoptotic efficacy of DBT evaluated by CCK-8 assay (a, b) A 4-hour CCK-8 assay was performed in 96-well plates to assess cell survival (450 nm absorbance measured at 0, 1, 2, 3, and 4 h). Blue light cycles consisted of 20 min irradiation (488 nm) followed by 40 min of dark incubation. Data were normalized to the 0 h value of each well, and outliers were excluded from analysis. Statistical significance was determined using a two-way ANOVA (mixed-effects model, n = 16 per group). Analysis of the main group effect revealed no significant differences under dark incubation (p = 0.4947). Under blue light, DBT treatment maintained cell viability, demonstrating anti-apoptotic effects (****p < 0.0001; ***p = 0.0006). In contrast, HBT induced apoptosis, resulting in reduced cell viability compared to CTRL and DBT: 1 h (ns, p = 0.1469; ****p < 0.0001), 2 h (ns, p > 0.9999; *p = 0.0145), 3 h (ns, p = 0.9998; *p = 0.0327), and 4 h (***p = 0.0006; ****p < 0.0001).

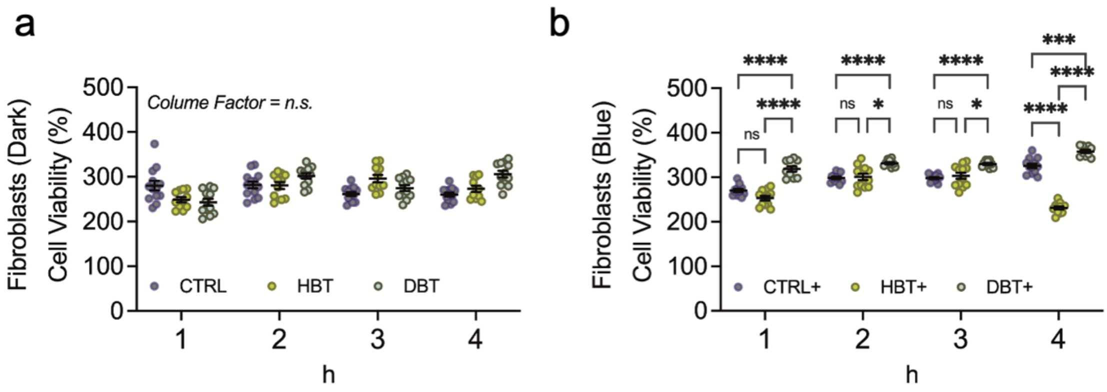

Supplementary Figure 23. Comparison of anti-apoptotic efficacy between DBT and BAI1 A 4-hour CCK-8 assay was performed to evaluate the efficacy of BAI1 treatment. Fibroblasts were pre-treated with 50 μM BAI1 for 1 h prior to the experimental onset. Blue-light cycles consisted of 20 min irradiation (488 nm) followed by 40 min of dark incubation. Absorbance at 450 nm was measured hourly (0–4 h). Data were normalized to the 0 h baseline, and outliers were excluded ($n = 16$ per group). Statistical significance was determined using a two-way ANOVA (mixed-effects model) with Dunnett's multiple comparisons test. (a) Under dark incubation, no significant main effect of group was observed ($p = 0.4852$). (b) Under blue-light irradiation, DBT maintained significantly higher viability throughout the 4-hour period (****$p < 0.0001$ for all). In contrast, HBT+BAI1 exhibited progressively reduced viability: 1 h (ns, $p = 0.1383$; ****$p < 0.0001$), 2 h (ns, $p = 0.0719$; **$p = 0.0051$), 3 h (ns, $p = 0.1271$; **$p = 0.0074$), and 4 h (****$p < 0.0001$). (c) Direct comparison of DBT with BAI1-treated controls (CTRL+BAI1) and HBT+BAI1 indicated that DBT conferred consistently superior protection at 1–4 h (****$p < 0.0001$ for all; *$p = 0.0104$; **$p = 0.0030$; ***$p = 0.0004$).

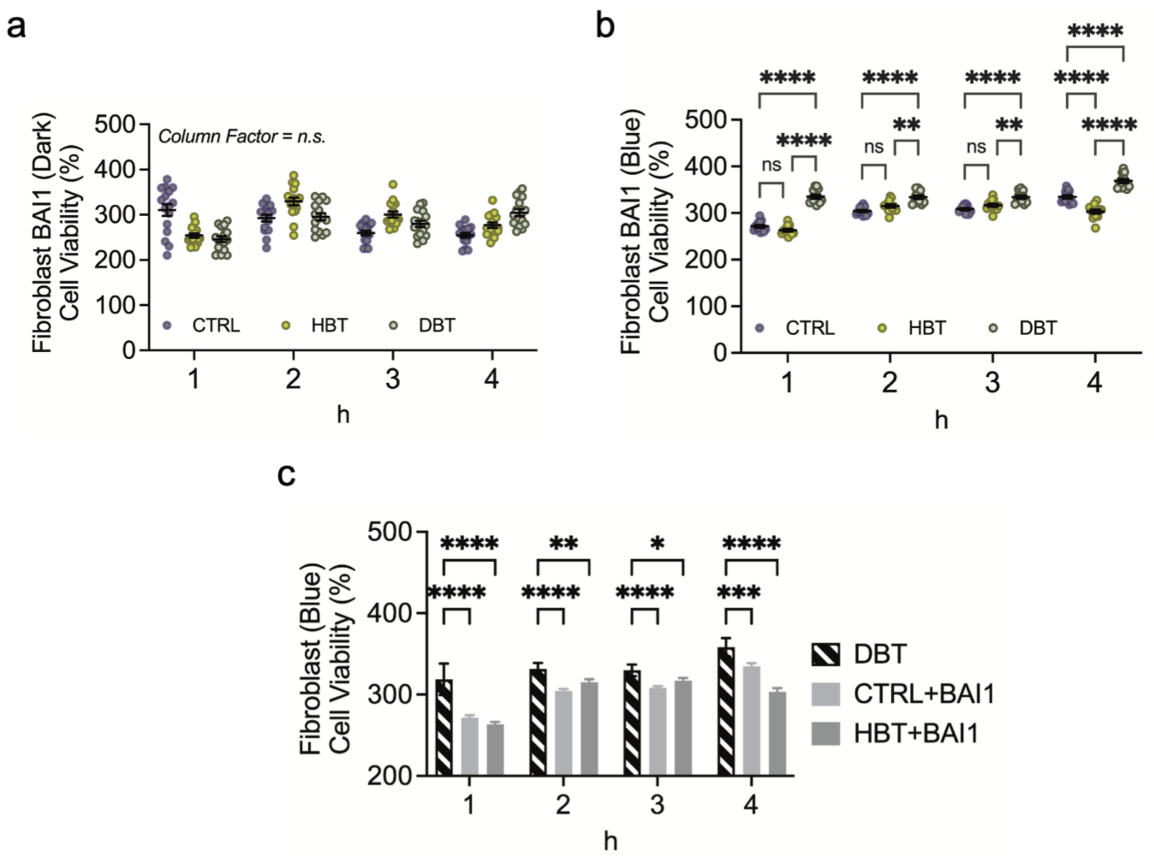
a
Fibroblast BAI1 (Dark)
Cell Viability (%)
Column Factor = n.s.
CTRL
HBT
DBT
h
b
Fibroblast BAI1 (Blue)
Cell Viability (%)
ns
****
**
***
CTRL
HBT
DBT
h
c
Fibroblast (Blue)
Cell Viability (%)
DBT
CTRL+BAI1
HBT+BAI1
h

Supplementary Figure 24. Protective effect of DBT against TNF-α–induced apoptosis under blue-light activation A 4-hour CCK-8 assay was performed to evaluate the efficacy of DBT against TNF-α-induced extrinsic apoptosis. Fibroblasts transfected with CTRL, HBT, or DBT constructs were treated with recombinant TNF-α (20 ng/mL) 1 h prior to assay. Absorbance (450 nm) was measured hourly (0–4 h). Light cycles consisted of 20 min irradiation (488 nm) followed by 40 min of dark incubation. Data were normalized to the 0 h baseline, excluding outliers (n = 16 per group). Statistical significance was determined using a two-way ANOVA (mixed-effects model) with Dunnett's multiple comparisons test. (a) Under dark incubation, no significant main effect of group was observed ($p = 0.3381$). (b) Under blue-light irradiation, significant differences were observed at 1 h (*$p = 0.0162$), 2 h (*$p = 0.0277$; ns, $p = 0.8197$; ***$p = 0.0003$), 3 h (*$p = 0.0303$; ns, $p = 0.9632$; **$p = 0.0012$), and 4 h (**$p = 0.001$; *$p = 0.0152$), with a significant overall group effect (****$p < 0.0001$). DBT conferred significantly higher viability compared to TNF-α–treated controls and HBT cells. Although differences compared to HBT varied at specific time points, likely reflecting cell-cycle dynamics, DBT consistently maintained higher overall viability. These findings demonstrate that DBT effectively counteracts TNF-α–induced apoptosis in a light-dependent manner.

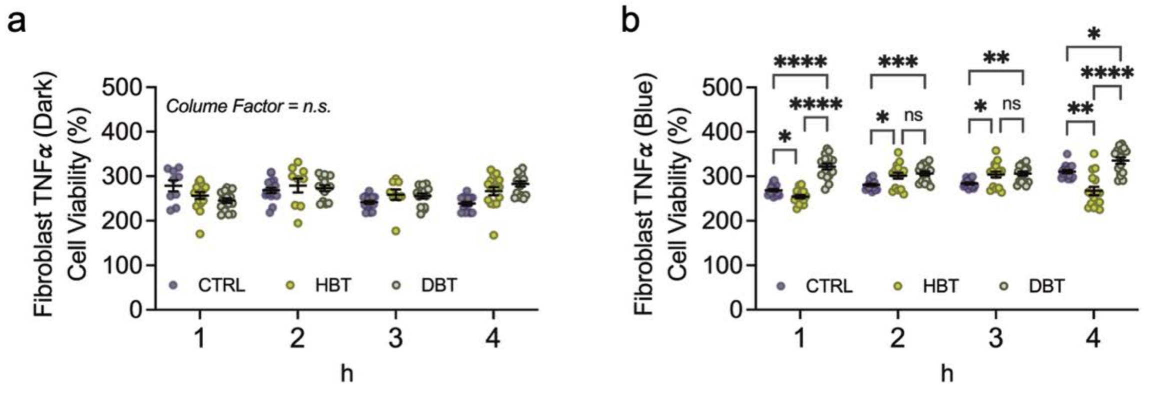

Supplement: Supplementary file 1 — Supplementary Information [file 12276_2025_1605_MOESM1_ESM.pdf]
